# Scaling relations of CO_2_ hydrogenation and dissociation on single metal atom doped In_2_O_3_ catalysts with promoted oxygen vacancy sites†

**DOI:** 10.1039/d4ra09111f

**Published:** 2025-03-12

**Authors:** Yuanjie Bao, Ziqi Tang, Yuchen Wang, Shenggang Li

**Affiliations:** a CAS Key Laboratory of Low-Carbon Conversion Science and Engineering, Shanghai Advanced Research Institute, Chinese Academy of Sciences 100 Haike Road Shanghai 201210 P. R. China lisg@sari.ac.cn; b University of Chinese Academy of Sciences Beijing 100049 P. R. China; c School of Physical Science and Technology, ShanghaiTech University Shanghai 201210 P. R. China; d State Key Laboratory of Low Carbon Catalysis and Carbon Dioxide Utilization, Shanghai Advanced Research Institute, Chinese Academy of Sciences Shanghai 201210 P. R. China

## Abstract

In this work, we conducted a computational study on single atom doped In_2_O_3_ catalysts with 12 transition metals (Fe–Cu, Ru–Ag, Os–Au) through density functional theory (DFT) calculations, by investigating the dissociation of H_2_, and the dissociation and hydrogenation of CO_2_. From the thermodynamic-kinetic scaling relationships such as Brønsted–Evans–Polanyi (BEP) and transition-state scaling (TSS) relations, we establish the descriptors for the energy barriers and improve our understanding of the synergistic catalytic effect of oxygen vacancies and single atoms. We find that the adsorption energy of the H adatom on the perfect surface can serve as an effective descriptor for the dissociation energy barrier of H_2_ on this surface, and the formation energy of the oxygen vacancy can serve as an effective descriptor for the energy barrier of CO_2_ hydrogenation to HCOO as well as the energy barrier of CO_2_ direct dissociation.

## Abstract 2

Oxygen vacancy formation energy can serve as an effective descriptor for CO_2_ hydrogenation to HCOO and CO_2_ dissociation to CO.

## Graphical abstract

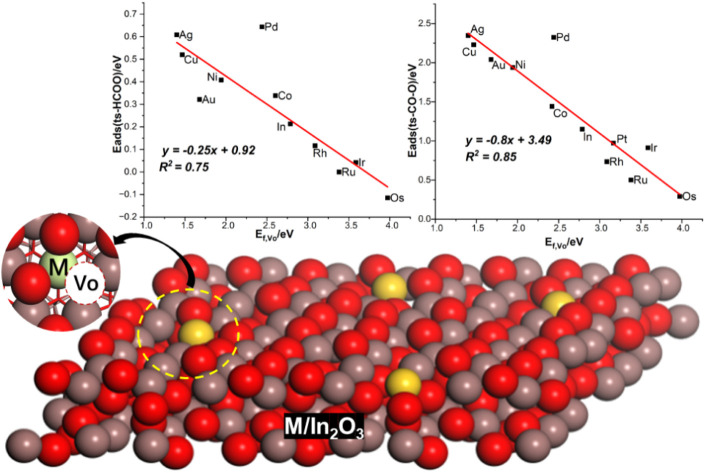

## 1. Introduction

To address the severe environmental issues caused by excessive carbon emissions, technologies for carbon capture, utilization and storage (CCUS) have gained widespread attention.^1–3^ Much effort has been made for the exploration of the CO_2_ hydrogenation to methanol reaction^4,5^ with the aim of simultaneously improving the utilization of renewable energy sources. At present, methanol synthesis at an industrial scale relies much on the conversion of synthesis gas, which is a mixture of CO and H_2_ with a small amount of CO_2_ facilitated by the Cu/ZnO/Al_2_O_3_ catalysts. Nonetheless, Cu-based catalysts are notably active for the reverse water–gas shift (RWGS) reaction, leading to lower methanol selectivity and catalyst deactivation especially at relatively high reaction temperatures.^6–8^

In_2_O_3_ has been regarded as a highly promising catalyst for CO_2_ hydrogenation to methanol in recent years.^9,10^ Interestingly, its potential was initially unveiled through density functional theory (DFT) calculations by Ye *et al.*,^11,12^ which has been confirmed through follow-up experiments.^13^ Structural characterization of the In_2_O_3_ catalyst by Frei *et al.*^14^ evidenced a dominant exposure of the (111) facet, and the CO_2_ hydrogenation reaction was considered to proceed *via* the selective and consecutive addition of hydrides and protons. Such a viewpoint was also supported by the DFT calculations of Qin *et al.*,^15^ indicating that the heterolytic dissociation of H_2_ at surface In and O pair sites is kinetically favorable on both the perfect and defective In_2_O_3_ surfaces. Martin *et al.*^16^ experimentally showed that methanol selectivity could reach 100% using the In_2_O_3_/ZrO_2_ mixed-oxide catalyst under the industrially relevant conditions (*T* = 473–573 K, *P* = 10–50 bar, GHSV = 16 000–48 000 h^−1^). Dang *et al.*^17^ found that the threefold oxygen vacancy site on the cubic c-In_2_O_3_(111) and hexagonal h-In_2_O_3_(104) surfaces favored the linear CO_2_ physisorption structure and the HCOO pathway, leading to high CH_3_OH selectivity.

While In_2_O_3_ allows for high methanol selectivity by effectively suppressing the rival RWGS reaction, CO_2_ reactivity is hindered by its relatively low activity for the dissociation of molecular H_2_.^18^ To enhance hydrogen activation, a range of metal promoters has been investigated including Pd, Pt, Ag, Ru, Rh, Ir, Ni, Re, and Au.^19–27^ Several of these studies indicate that highly dispersed metal promoters play a crucial role in enhancing the catalytic activity of In_2_O_3_ for the methanol synthesis reaction. To facilitate a direct comparison of the formation and promotional effects of these catalysts, Pérez-Ramírez *et al.*^28^ introduced 9 metal promoters into In_2_O_3_ at the same loading of 0.5 wt% through flame spray pyrolysis (FSP) as a standardized synthesis method. It was found that atomically dispersed promoters such as Pd, Pt, Rh, Ru and Ir led to the greatest performance improvement, especially Pd and Pt, which significantly promote hydrogen activation while hindering CO formation. Shen *et al.*^27^ further demonstrated that at low Re loadings of ≤1 wt%, Re was doped into the In_2_O_3_ lattice in a single atom form, which benefits methanol formation. Huang *et al.*^29^ designed a bifunctional single atom catalyst (SAC) based on the synergy of atomic Ir and In_2_O_3_ and revealed that a Lewis acid–base pair site was formed between the atomic Ir and the adjacent oxygen vacancy (V_O_) site on In_2_O_3_ to form two distinct catalytic centers, which could reduce CO_2_ to the active intermediates and then facilitated the C–C coupling reaction to form ethanol.

The above studies demonstrate that the atomically dispersed M/In_2_O_3_ SAC is promising for the CO_2_ hydrogenation to methanol reaction. However, due to the difficulty in experimental preparation and characterization of single atoms, the structure–activity relationship of these atom-doped catalysts remains elusive. DFT calculations have been widely used in the field of catalysis for decades, typically for understanding experimental results, elucidating reaction mechanisms, establishing microkinetic models, and predicting structure–activity relationship.^30,31^ However, complex reaction networks as well as compositional complexity pose a significant challenge. To reduce the computational cost, linear correlations such Brønsted–Evans–Polanyi (BEP) and transition-state scaling (TSS) relations were investigated for the rapid estimation of energy barriers.^32,33^ The former reflects the relationship between the reaction energy and the energy barrier, whereas the latter suggests a linear relationship between the adsorption energy of the initial or final state and the energy barrier. These scaling relations reveal the factors that affect the catalytic activity of different materials, generally known as descriptors. Zhao *et al.*^30^ recently gave an overview of the reactivity descriptors for diverse catalytic systems, encompassing both electronic descriptors such as d-band center of metal and structural descriptors such as coordination numbers (CN) of the active site. It has been generally recognized that the development of effective scaling relationships and descriptors is vital for the rational design of catalytic systems.

Although single atom doped In_2_O_3_ catalysts serve as excellent theoretical models, there have been few researches on their scaling relations and descriptors. Chen *et al.*^34^ found the relationship between CO_2_ adsorption energies and the adsorption energies of transition states on 9 single-metal-atom-doped In_2_O_3_(110) surfaces. However, the formation energy of oxygen vacancy (*E*_f,V_O__) has not been explored as a possible descriptor, which has important influence on the adsorption and activation of CO_2_ based on previous studies.^12,35,36^ In addition, previous studies showed that there were differences in the methanol selectivity for different In_2_O_3_ facets and the (111) surface was the most stable surface under experimental conditions.^17^ Previous studies from our group^37^ found that the single atom-doped In_2_O_3_ surface can promote the formation of surface oxygen vacancies, thereby promoting the adsorption and activation of CO_2_ on the surface and triggering the subsequent RWGS reaction.

In this work, we performed extensive DFT calculations to explore the synergistic effect of single metal atom, oxygen vacancies, and In_2_O_3_ for the activations of H_2_ and CO_2_, where the metals were selected based on previous experiments.^28^ By regulating the formation energy of the oxygen vacancy through single metal atom doping, we aim to demonstrate the influence of oxygen vacancies and metal dopants on CO_2_ reactivity.

## 2. Computational details

DFT calculations were performed using the Vienna *ab initio* simulation package (VASP).^38,39^ The generalized gradient approximation (GGA) with the Bayesian error estimation functional including the van der Waals correction (BEEF-vdW)^40^ was employed to treat the electron exchange and correlation in the Kohn–Sham theory. The parameters used in this work are similar to those in our previous works.^15,17,26,37^ A plane wave energy cut-off of 400 eV and the Gaussian smearing width of 0.05 eV were employed. Convergence thresholds for the energy and force were set to 10^−4^ eV and 0.03 eV Å^−1^, respectively. Both the climbing image nudged elastic band (CI-NEB) method^41,42^ and the dimer method^43^ were used to find the transition states (TS), which were further confirmed through harmonic frequency analysis.

Similar to our previous work,^44^ the c-In_2_O_3_(111) surface was built from the optimized primitive unit cell and modeled with a p(1 × 1) slab consisting of 48 In atoms and 72 O atoms distributed in three O–In–O trilayers. The supercell has a dimension of 14.44 Å × 14.44 Å × 17.99 Å, where the bottom layer was fixed and a vacuum layer of 15 Å was inserted between adjacent slabs. The Brillouin zone was sampled using a (3 × 3 × 1) Monkhorst–Pack *k*-point mesh.^35,44^ For the Fe, Co and Ni-doped model, spin polarization was enabled.

Substitution of an In atom in the topmost layer of the c-In_2_O_3_(111) surface by a transition metal (M) atom (Fe–Cu, Ru–Ag, Os–Au) results in the model denoted as M/In_2_O_3_. The adhesive energy (Δ*E*_adh_) of the single metal atom is defined in eqn (1):

1 Δ*E*_adh_ = *E*_M/In_2_O_3__ − *E*_V_In_ − *E*_M_

where *E*_M/In_2_O_3__, *E*_V_In_, and *E*_M_ are the total energies of the surface with the In atom replaced by a single metal atom, that with the In atom removed, and the free single metal atom, respectively. The cohesive energy of metal (Δ*E*_coh,M_) is defined as the energy the metal atom in the condensed phase relative to that in the gas phase from eqn (2):

2 Δ*E*_coh,M_ = *E*_bulk,M_/*n*_M_ − *E*_M_

where *E*_bulk,M_ and *n*_M_ are the energy of the metal atom and the number of atoms in the bulk unit cell, respectively. The relative stability of the single metal atom can then be determined by calculating Δ*E*_stability_, which is defined in eqn (3):

3 Δ*E*_stability_ = Δ*E*_adh_ − Δ*E*_coh,M_

Definitions of the formation energy of a V_O_ site (Δ*E*_f,V_O__) and the adsorption energy of an adsorbate A on a slab surface denoted as *E*_ads_(A) are similar to our previous works.^15,45^ Briefly, Δ*E*_f,V_O__ was calculated as the reaction energy of the thermal desorption of molecular O_2_ from eqn (4):

4 Δ*E*_f,V_O__ = *E*_surface_V_O__ − *E*_perfect_ + 1/2 × *E*_O_2__

where *E*_surface_V_O__, *E*_perfect_ and *E*_O_2__ denote the total energies of the defective surface with a V_O_, the perfect surface, and the gas phase O_2_. *E*_ads_(A) is defined from eqn (5):

5 *E*_ads_(A) = *E*_total_ − (*E*_slab_ + *E*_A_)

where *E*_total_, *E*_slab_ and *E*_A_ are the total energies of the slab with the adsorbate, the clean slab, and the adsorbate as a free molecule, respectively. When the adsorbate A is adsorbed at an atomic site B on the surface, the adsorption energy is denoted as *E*_ads_(A@B); when the adsorbates A and C are adsorbed at two atomic sites B and D on the surface, the co-adsorption energy is denoted as *E*_ads_(A@B&C@D). All structures were built and visualized using the Materials Visualizer from the Materials Studio,^46^ and their optimized fractional coordinates are provided in the ESI.†

## 3. Results and discussion

### 3.1 Thermal stabilities and electronic structures of M/In_2_O_3_(111)

**Fig. 1:**
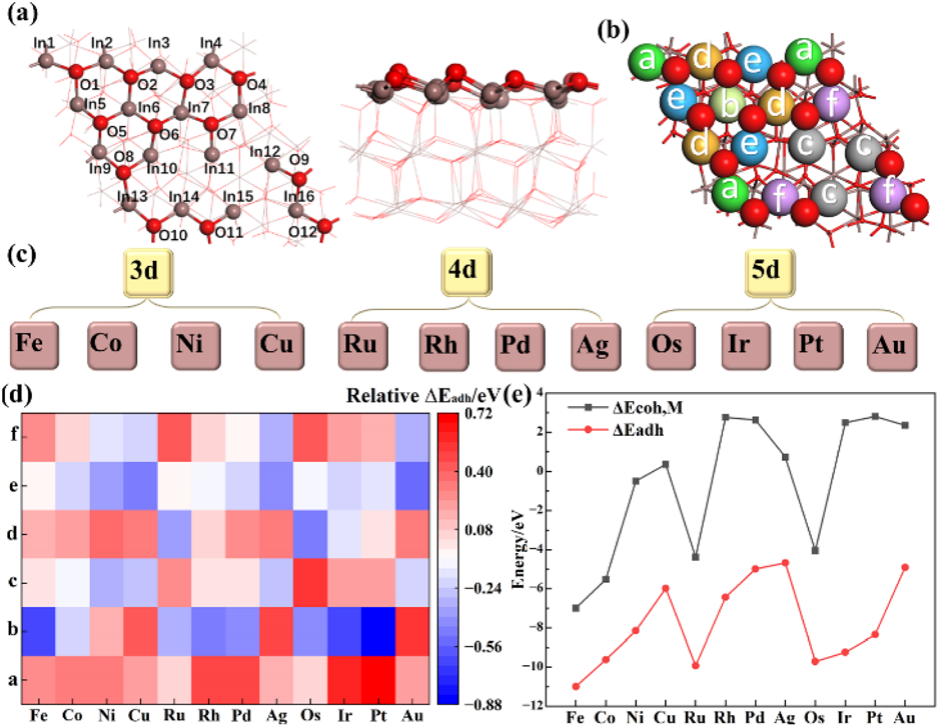
(a) Top and side views of the stoichiometric In_2_O_3_(111) surface with numbers of surface In (grey) and O (red) atoms, (b) different In sites for metal doping on the In_2_O_3_(111) surface, (c) selected transition metals for doping explored in this work, (d) relative Δ*E*_adh_ for single metal atom doped at the In_a_–In_f_ sites, (e) comparison between adhesive energies and cohesive energies of the metal dopant at the In_b_ site.

The model of In_2_O_3_(111) surface is shown in Fig. 1(a). Based on the coordination environments of the In atoms on In_2_O_3_(111) surface, they can be classified into six categories,^37^ namely In_a_–In_f_, as shown in Fig. 1(b). For the selected transition metals studied in this work as shown in Fig. 1(c), the adhesive energy of the metal dopant (Δ*E*_adh_) was calculated for all different In sites. As shown in Fig. 1(d), most of the single metal atom substitutions for the In_b_ site lead to the lowest energy among the different In sites, which is chosen for metal doping. Previous studies^26,28^ suggest that Pd, Pt, Rh, Ru, Ni and Ir can be atomically dispersed into In_2_O_3_ by co-precipitation and flame spray pyrolysis (FSP). To reveal the stability of the doped structure, Δ*E*_adh_ is compared with the binding energy of the single metal (Δ*E*_coh,M_) as shown in Fig. 1(e), and Δ*E*_adh_ is always more negative than Δ*E*_coh,M_, indicating a stronger interaction between the single metal atom and the In_2_O_3_(111) surface than that between the single metal atoms, which may prevent the aggregation of the single metal atoms. Values of the calculated Δ*E*_stability_ for all doped surfaces are shown in Table 1, where more negative values indicate stronger interaction between the single metal atom and the In_2_O_3_(111) surface than that between the single metal atoms.

**Table 1:** Δ*E*_stability_ (eV) calculated for all doped surfaces

Surface	Fe/In_2_O_3_	Co/In_2_O_3_	Ni/In_2_O_3_	Cu/In_2_O_3_
Δ*E*_stability_	−4.00	−4.09	−7.67	−6.36
Surface	Ru/In_2_O_3_	Rh/In_2_O_3_	Pd/In_2_O_3_	Ag/In_2_O_3_
Δ*E*_stability_	−5.55	−9.20	−7.61	−5.41
Surface	Os/In_2_O_3_	Ir/In_2_O_3_	Pt/In_2_O_3_	Au/In_2_O_3_
Δ*E*_stability_	−5.66	−11.73	−11.16	−7.25

As shown in Fig. S1(a),† based on the coordination environment the surface oxygen atoms can be classified into four categories (O_a_–O_d_) when the single metal dopant is at the In_b_ site. For a better illustration of their interaction, the atoms on the In_2_O_3_(111) surface are shown by the 2 × 2 supercell in Fig. S1(b).† The charge depletion of the single atoms at the In_b_ site is reduced compared to the pristine In_2_O_3_(111) surface as shown in Table 2, suggesting a lower valence doping, consistent with previous experimental observations.^26,28^ Differential charge density analysis shown in Fig. S1(c)† indicates that most of the charge redistributions are concentrated in the single metal atom and adjacent In and O atoms, although there are slight charge redistributions among other surface and subsurface atoms, which are further confirmed by our Bader charge analysis as listed in Table S1.† The average Bader charges of the O_a_, O_b_, O_c_ and O_d_ sites are −1.18, −0.97, −1.15 and −1.16|*e*| for the single atom doped surfaces, compared to those of −1.17, −1.15, −1.15 and −1.17|*e*| on the pristine In_2_O_3_ surface, indicating that the charge reduction of the single atoms decreases the charge of adjacent O_b_ atoms directly bound to the single metal atoms.

**Table 2:** Bader charges carried by the single metal atom (M) on the clean surface, the surface with an H adatom at the O_b_ site, and the defect surface

Surface	*q*(M)/|*e*|		
	Clean surface	H-Adsorbed surface	Defect surface
Fe/In_2_O_3_	1.48	1.42	1.33
Co/In_2_O_3_	1.34	1.30	1.21
Ni/In_2_O_3_	1.29	1.17	1.06
Cu/In_2_O_3_	1.18	1.09	1.03
Ru/In_2_O_3_	1.58	1.45	1.29
Rh/In_2_O_3_	1.29	1.22	1.11
Pd/In_2_O_3_	1.28	1.10	0.79
Ag/In_2_O_3_	1.06	0.90	0.71
Os/In_2_O_3_	1.85	1.70	1.51
Ir/In_2_O_3_	1.51	1.40	1.21
Pt/In_2_O_3_	1.40	1.41	0.81
Au/In_2_O_3_	1.04	1.01	0.99
In_2_O_3_	1.89	1.83	1.72

In addition, there is an apparent linear relation between the Bader charge of the single atom (*q*(M)) and the formation energy of the O_b_ vacancy as shown in Fig. S1(d),† indicating the single metal atom can affect the formation of the adjacent oxygen vacancy through charge transfer.

### 3.2 Scaling relations for H_2_ dissociative adsorption on the perfect surface

**Fig. 2:**
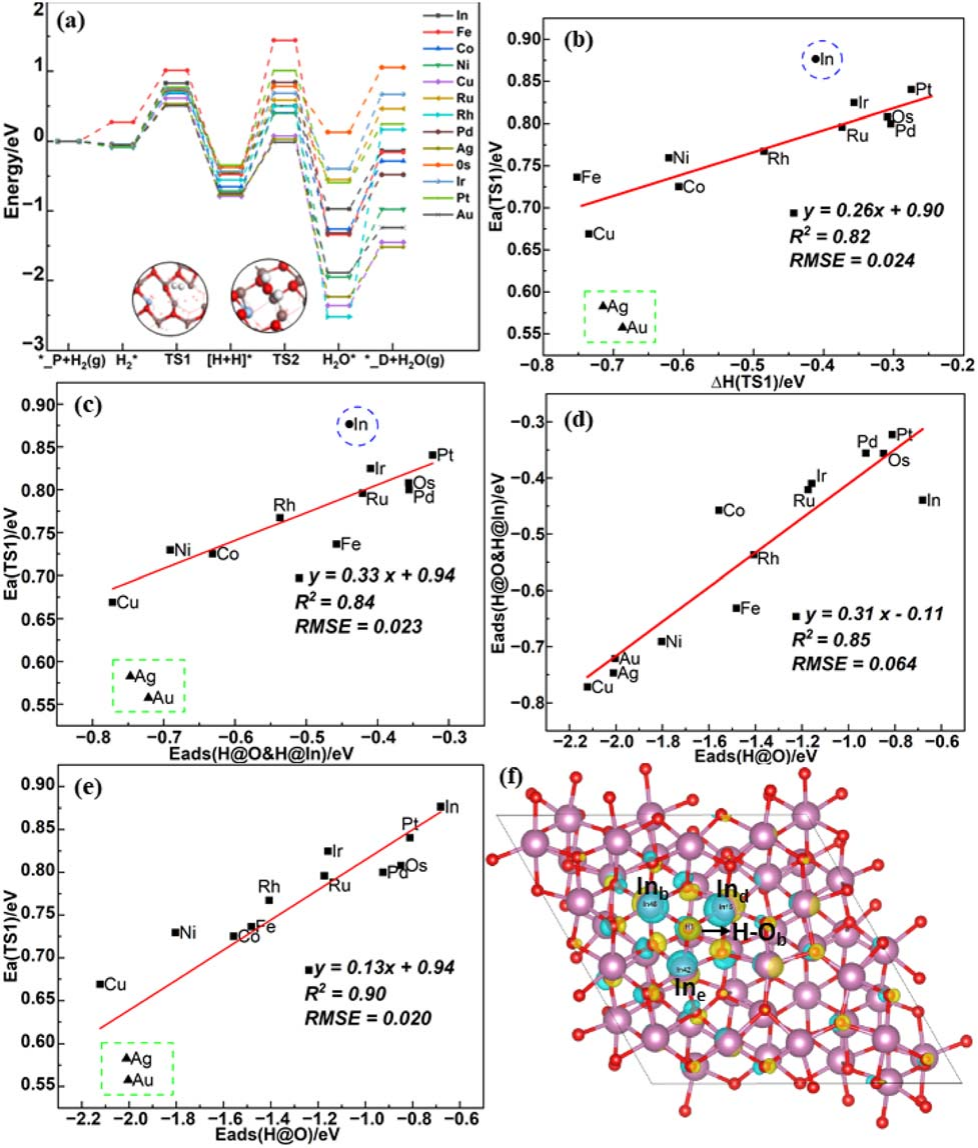
(a) Energy profiles of H_2_ heterolysis and V_O_ formation on the perfect surfaces, (b) BEP relation of H_2_ heterolysis (TS1), (c) TSS relation of H_2_ heterolysis (TS1), (d) scaling relation between *E*_ads_(H@O) and *E*_ads_(H@O&H@In), (e) scaling relation between *E*_ads_(H@O) and energy barrier of TS1, (f) the differential charge density of H adsorption surface, in which light blue and yellow regions indicate charge accumulation and depletion, respectively.

Previous studies^14,47^ suggested that heterolytic dissociation of H_2_ that leads to a proton bound to an O atom and a hydride bound to an In atom is easier than homolytic dissociation on the In_2_O_3_(111) perfect surface. Due to the lower stability of H adsorbed at the M site on the doped surface, it is easy for the H adatom to migrate to the surrounding oxygen, making it less likely to form the H@M&H@O pair (H@M&H@O refers to co-adsorption of H on M and H on O). Thus, only the H@In&H@O pair is considered. The potential energy surface of H_2_ heterolysis and further water formation is shown in Fig. 2(a).

H_2_ heterolysis is thermodynamically favorable but on the pristine In_2_O_3_ surface incurs a modest energy barrier of 0.88 eV. Single metal doping reduces this energy barrier to 0.56–0.84 eV. In addition, Au, Ag and Cu doping are more favorable for H_2_ dissociation with lowest energy barriers among the studied elements in the same transition row. The subsequent formation of H_2_O by the transfer of the hydride from In_e_–H to the adjacent hydroxyl group (O_b_–H) on most single metal atom doped In_2_O_3_ surfaces incurs a higher energy barrier ranging from 1.02 to 1.92 eV than that on the pristine In_2_O_3_ surface of 0.96 eV, whereas Au, Ag and Cu doping can promote oxygen vacancy formation because of their much lower energy barrier (from 0.73 to 0.87 eV).

In addition, Table S2† further shows that the adsorption energy of two H adatoms as hydroxyls (−3.7 eV) is much higher than that of H@In&H@O (−0.77 eV) for Ag/In_2_O_3_, making it difficult to break one of the O–H bonds to form H_2_O, consistent with a higher energy barrier of 1.49 eV than that to H_2_O *via* H@In&H@O of 0.80 eV as shown in Table S3,† suggesting that the In_2_O_3_ surface may be hydroxylated under the typical reaction conditions.^14^ Contrary to the work of Pérez-Ramírez *et al.*^28^ we find that oxygen vacancy formation by H_2_ reduction is easier for Ag/In_2_O_3_ and Au/In_2_O_3_ due to their relatively low energy barriers when they are atomically dispersed on the In_2_O_3_(111) surface. However, in their experiments, Ag and Au may actually interact with the In_2_O_3_ catalyst in the form of metal clusters, so their effect on oxygen vacancy formation may differ from our theoretical predictions.

BEP relations are widely studied for the activation of gas phase species (such as H_2_) on surfaces of transition metals and their oxide.^31^ As shown in Fig. 2(b), the BEP relation of H_2_ dissociation on all doped surfaces is obvious except for Au and Ag doped surfaces and the pristine In_2_O_3_ surface. This may be due to that the transition state structures for these surfaces differ significantly from their final state structures, as also noted by a previous study.^33^ As shown in Table S4,† their In–H bond lengths (2.37 and 2.45 Å) are significantly longer than those of the other surfaces (1.98–2.23 Å). Owing to the weak physisorption of H_2_ in the initial state, the reaction energy is close to the adsorption energy of the final state, and the transition state scaling (TSS) relation shown in Fig. 2(c) reveals that the adsorption energy of the final state rather than that of the initial state has a more significant impact on the transition state energy. Besides, the adsorption energy of the H adatom at the In_e_ site (*E*_ads_H@In) on the different surfaces remains basically unchanged as shown in Table S2,† so the adsorption energy of the final state is mainly determined by the adsorption energy of H on O (*E*_ads_H@O) with a coefficient of determination (*R*^2^) of 0.85 as shown in Fig. 2(d). This is consistent with the good linear relation between *E*_ads_H@O and the energy barrier with an *R*^2^ value of 0.90 as shown in Fig. 2(e).

From the differential charge density analysis of H adsorption on the surface in Fig. 2(f), electron transfer mainly occurs between O_b_ and the atoms directly bound to it. The changes in the charge of the single metal atom and the O_b_ atom on the clean and H-adsorbed surfaces shown in Table 2 indicate that the charge carried by the single metal atom decreases while the number of electrons acquired by the O_b_ atom increases, suggesting that charge transfer occurs between the single metal atom, the O_b_ atom and the H adatom. Furthermore, *E*_ads_H@O scales linearly with the charge carried by the single metal atom (*q*(M)) as shown in Fig. S2(a),† indicating that the reactivity of the adjacent oxygen site is enhanced because of the higher *E*_ads_H@O than that on the pristine In_2_O_3_ surface. Besides, *E*_ads_H@O also scales linearly with the p-band center of the O site (*ε*_p_) with a negative slope in Fig. S2(b),† which was previously proposed as a descriptor for the adsorption energies of intermediates involved in the oxygen evolution reaction (OER) reaction on perovskite surfaces.^30^ In addition, here we construct a descriptor *φ* by combining the effects of both *ε*_d_ and *ε*_p_ through a multivariate linear regression model as shown in eqn (6):

6 *φ* = −0.1 × *ε*_d_ − 0.77 × *ε*_p_ − 2.73

As shown in Fig. S2(c and d),† there is a linear relation between the energy barrier of TS1 or *E*_ads_H@O and the combined value of the O p-band center (*ε*_p_) and M d-band center (*ε*_d_). The reason for considering *ε*_d_ is that electrons from the H adatom are transferred to the O_b_ atom, leading to their subsequent transfer into a vacant d-orbital of the single metal atom on the H-adsorbed surfaces.^48^ The initial, transition and final state structures for TS1 are shown in Fig. S3(a).† Upon water desorption, the surface oxygen vacancy is formed as shown in Fig. S3(b).†

### 3.3 Scaling relations for H_2_ dissociative adsorption on defective In_2_O_3_ surfaces

**Fig. 3:**
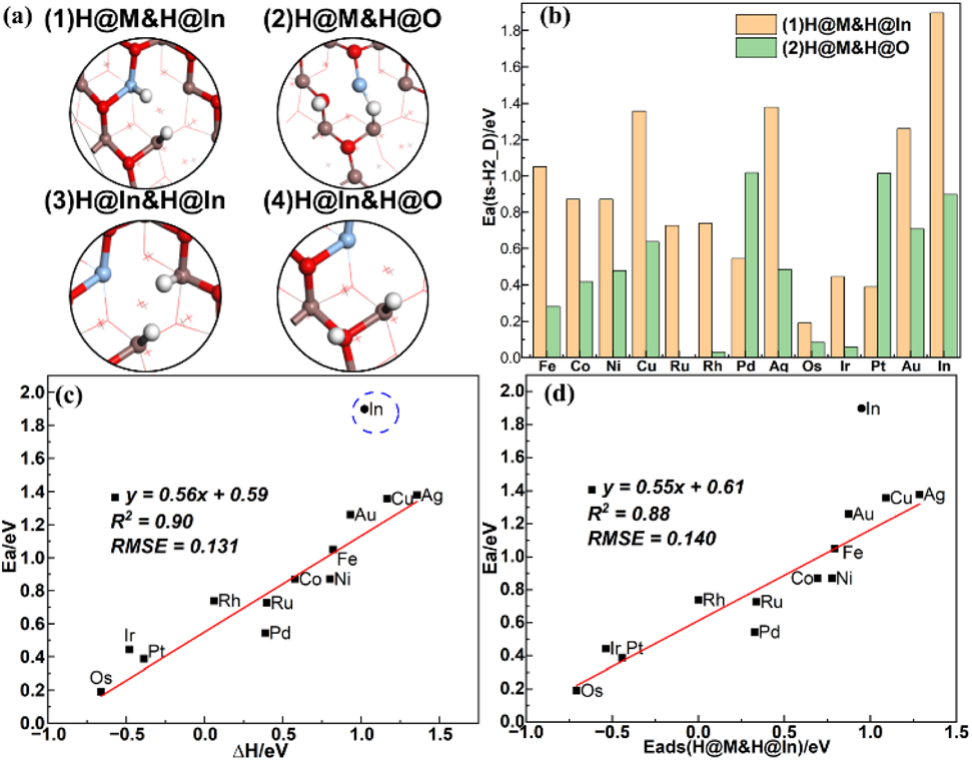
(a) Different final state structures from the various possible pathways of H_2_ dissociation, (b) comparison of the energy barriers of pathways (1) and (2), (c) BEP relation of pathway (1), (d) TSS relation of pathway (1).

As shown in Fig. 3(a), the dissociative adsorption of H_2_ at the V_O_ site on the defect In_2_O_3_ surfaces can occur *via* four possible pathways leading to the formation of (1) H@M&H@In, (2) H@M&H@O, (3) H@In&H@In, and (4) H@In&H@O (with the H adsorbed at the top site). Our previous work^47^ shows that H_2_ dissociation to form H@In&H@O has a lower energy barrier than that to H@In&H@In. As shown in Table S5,† the adsorption energies of H@In&H@O are similar for all model surfaces and the adsorption energies are approximately 0 eV for the initial state, leading to similar reaction energies (ΔH), so the BEP relation of H_2_ dissociation to form H@In&H@O may be untenable. This is demonstrated by our calculations for several model surfaces, which yield nearly the same energy barriers and reaction energies as shown in Table S6,† so no further calculations are performed for the pathway leading to H@In&H@O. As for pathway 1, 2 and 4, their initial, transition and final state structures on Ag doped defect surface are shown in Fig. S4.†

H_2_ dissociation *via* pathway (1) starts from H_2_ physisorption, and is endothermic on all doped surfaces except for Pt, Os and Ir, resulting in two hydrides both with negative charges. The energy barriers for this pathway on all defect surfaces are shown in the Fig. 3(b), ranging from 0.19 to 1.90 eV. The energy barriers of all defect doped surfaces are lower than the defect undoped surface, and the energy barriers of the defect Au, Ag and Cu doped surfaces are the highest among the elements in the same period, contrary to that on the perfect surfaces. Furthermore, H_2_ dissociation is easy to occur on the defect Os/In_2_O_3_ surface with a low energy barrier of 0.19 eV, and different from the transition state structure for the other doped surfaces, both H adatoms binds the Os sites and the final state is highly stable with an adsorption energy of −0.66 eV. The BEP relation in Fig. 3(c) with a *R*^2^ of 0.90 indicates that the transition state structures are more similar to each other except for the defect undoped surface, where one H adatom is located at the bridge site between In_b_ and In_d_ in its final state. As the adsorption energy of the initial state is approximately zero, there is a good TSS relation between the energy barrier and the adsorption energy of the final state as shown in Fig. 3(d). The high energy barriers of the defect Au, Ag and Cu doped surfaces are due to the high endothermic adsorption energies in the final state as shown in Table S7.† Due to the fact that single metal atom doping does not notably affects the adsorption energy of the H adatom at the In site (*E*_ads_H@In) as shown in Table S5,† the relative energy of the final state has a good linear relation with the adsorption energy of the H adatom at the single metal atom site (*E*_ads_H@M) as shown in Fig. S5(a),† which can serve as a descriptor for the energy barrier of H_2_ dissociation on the defect surfaces. The dissociative adsorption of H_2_ is more likely to occur with a reduced *E*_ads_H@M.

Previous studies suggest that the d-band center of surface metal site can affect the adsorption energy of the H adatom on transition metals and their oxides.^49^ However, our study shows that the d-band center does not scale linearly with the adsorption energy of the H adatom at the single metal atom site as shown in Fig. S5(b).† We attribute this to the presence of the oxygen vacancy, as the H adatom adsorbed at the top site tends to shift towards the oxygen vacancy, leading to the inclination of both the H–M and H–In bonds. The linear relation shown in Fig. S5(c)† indicates that adsorption of the H adatom at the single metal atom site is enhanced as the oxygen vacancy formation energy increases, which also scales linearly with the energy barrier of H_2_ dissociation as shown in Fig. S5(d).†

As shown in Fig. 3(b), the energy barriers of H_2_ dissociation *via* pathway (2) range from 0.00 to 1.02 eV. This pathway is kinetically more favorable than pathway (1) for all doped surfaces except for Pt and Pd doping. The energy barriers on the defect Pt/In_2_O_3_ and Pd/In_2_O_3_ are the highest, nearly the same as that on the undoped In_2_O_3_. Because H tends to adsorbs at the bridge site between M and In_e_ in the final state, resulting in a less stable structure than the top site of Pt and Pd after H_2_ heterolysis. However, on the Ru, Rh, Os and Ir doped surfaces, the energy barrier for H_2_ heterolysis is lower than 0.1 eV. In addition, no BEP or TSS relation is found in H_2_ dissociation *via* pathway (2) due to the significant differences in the transition state structures for all defect doped surfaces. Previous studies^17,27,34^ suggest that for CO_2_ hydrogenation, the hydrogen comes from the hydride (H–In), and our results show that H_2_ dissociation through M–O is more favorable, which may provide a different hydrogen source (H–M).

### 3.4 CO_2_ hydrogenation and dissociation on defect surfaces

Previous studies suggest that CO_2_ hydrogenation to methanol on the defect In_2_O_3_ surface occurs *via* the formate route 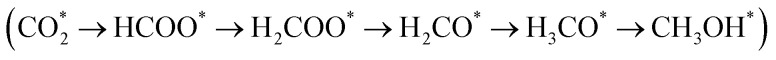, where the HCOO* specie is the key intermediate during methanol formation from early studies,^12,14,15^ whereas CO is formed *via* the RWGS route 
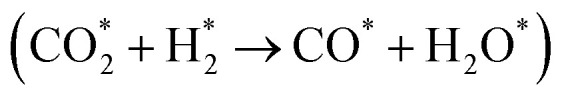
 initiated by CO_2_ protonation to COOH or CO_2_ direct dissociation 
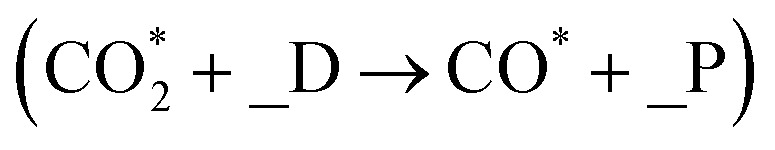
, where _D refers to the defect surface with V_O_ and _P refers to the perfect surface. Thus, the initial conversion of CO_2_ plays an important role in methanol synthesis activity, and the energy barriers of the elementary reactions involved in the initial conversion of CO_2_ on all doped surfaces are calculated to reveal the effect of metal doping on the CO_2_ conversion route, considering that the structure of the single metal atom doped In_2_O_3_(111) surface is similar to that of the pure In_2_O_3_(111) surface.

According to our previous study, there are two distinct CO_2_ adsorption configurations at the V_O_-b site on the defective (111) surface, namely the linear CO_2_
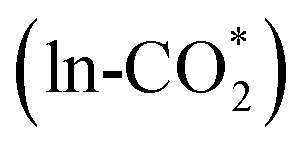
 and bent CO_2_
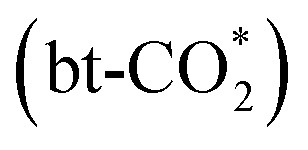
.^17^ For 
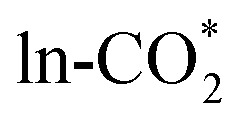
, the C atom is far from the M atom on all surfaces, both the C–O bond lengths are approximately 1.18 Å on all surfaces, which are close to the C–O bond lengths in gas CO_2_ and nearly no charge transfer occurs between the adsorbate and surface, indicating that CO_2_ weakly physisorbs above the O_b_ vacancy site. As shown in Table S8,† the adsorption energy of 
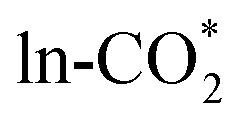
 ranges from −0.48 to −0.27 eV on the different surfaces, which is more negative than that from our previous work due to the inclusion of the van der Waals correction *via* the BEEF-vdW exchange–correlation functional. The bond length, bond angle, and adsorption energy of 
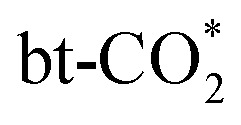
 are shown in Table 3. No stable structures were found for the 
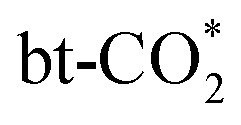
 adsorption configuration on the Ag, Au doped and pristine In_2_O_3_ surfaces. For the 
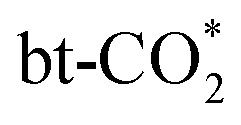
 adsorption configuration, the C–M bond length ranges from 1.99 to 2.17 Å, the C–O1 bond length ranges from 1.29 to 1.39 Å, while the C–O2 bond length ranges from 1.19 to 1.22 Å, and the O–C–O angle ranges from 120.6 to 143.4°, where O1 occupies the V_O_ and O2 only binds the C. There is significant charge transfer between CO_2_ and the surface, as also indicated by the differential charge density analysis shown in Fig. S6.† Thus, the bt-CO_2_ adsorbate is chemisorbed and activated with an adsorption energy ranging from −1.09 to 0.14 eV. This chemisorption is endothermic for Fe/In_2_O_3_ and Co/In_2_O_3_, whereas it is more exothermic on Os/In_2_O_3_, Ir/In_2_O_3_ and Pt/In_2_O_3_ than other catalysts. The adsorption energies of the H adatom, the linear and bent CO_2_ and their co-adsorption are given in Table S8.†

**Table 3:** Bond lengths/angles (O1–C–O2) and adsorption energies of 
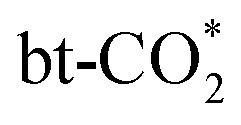
 where O1 occupies the V_O_ and O2 only binds the C

Surface	C–M/Å	C–O1/Å	C–O2/Å	Angle (O1–C–O2)/°	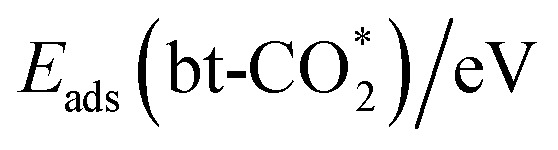
Fe/In_2_O_3_	2.17	1.29	1.19	134.7	0.14
Co/In_2_O_3_	1.99	1.34	1.20	127.3	0.11
Ni/In_2_O_3_	2.07	1.29	1.19	134.3	−0.03
Cu/In_2_O_3_	2.07	1.25	1.19	143.4	−0.08
Ru/In_2_O_3_	2.01	1.37	1.22	122.7	−0.55
Rh/In_2_O_3_	2.00	1.37	1.21	123.3	−0.45
Pd/In_2_O_3_	2.10	1.30	1.20	132.9	−0.32
Os/In_2_O_3_	2.03	1.39	1.22	120.6	−1.09
Ir/In_2_O_3_	2.01	1.39	1.21	121.3	−0.92
Pt/In_2_O_3_	2.05	1.34	1.20	126.3	−0.78

**Fig. 4:**
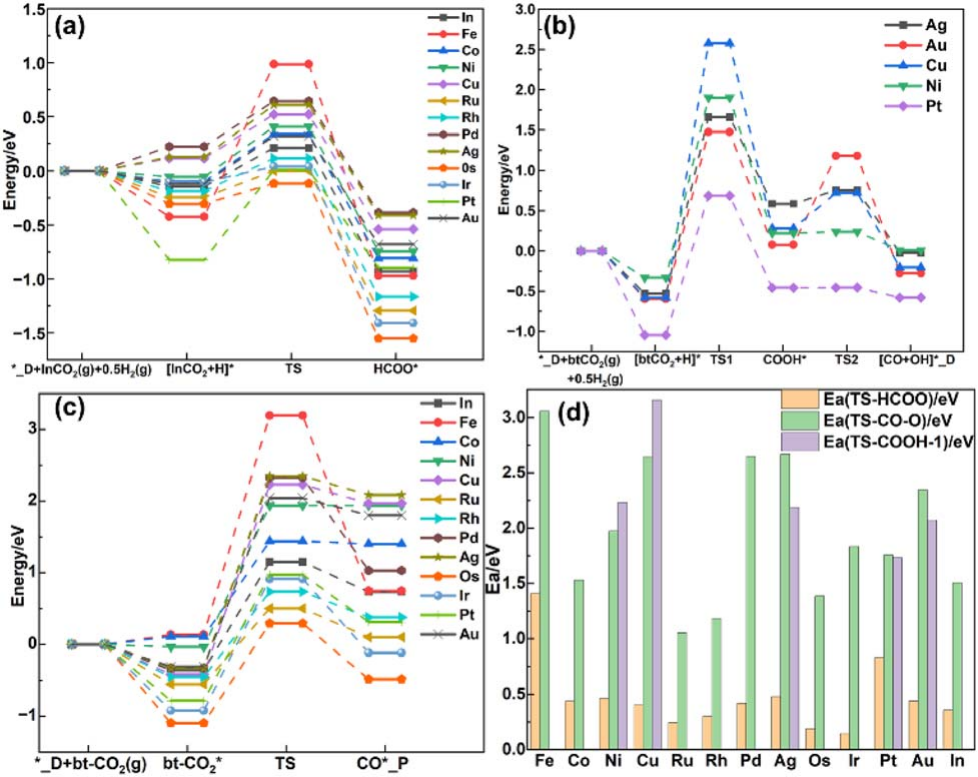
Energy profiles of (a) 
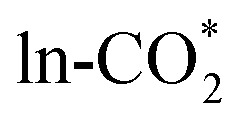
 hydrogenation to HCOO*, (b) 
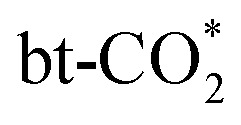
 protonation to COOH* and dissociation to CO* + OH*, (c) 
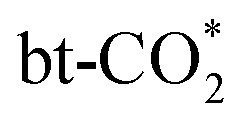
 dissociation to CO* + O*; (d) energy barriers of these three routes (TS-COOH-1 denotes 
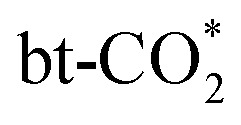
 protonation to COOH*).

In addition, for single Ni atom doped surface, Cannizzaro *et al.*^50^ previously predicted CO_2_ hydrogenation from the H adatom at the O site to have an energy barrier of 1.70 eV, while our group's previous calculation^51^ found a much lower energy barrier of 0.83 eV for CO_2_ hydrogenation from the H adatom at the In_e_ site. Here, we calculated and compared two different hydrogenation pathways on Ag, Ni, Os, Ir, and Pd doped In_2_O_3_ surfaces as listed in Table S9.† Two types of hydrides, namely H–In and H–M hydrides, are formed by H_2_ dissociation as mentioned in Section 3.3. HCOO* is formed by 
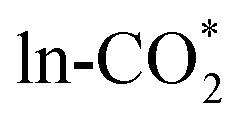
 hydrogenation with a hydride *via* the Eley–Rideal mechanism,^52^ where one O of HCOO* fills the oxygen vacancy as shown in Fig. S7.† Our calculations indicate that transfer of the H–M hydride has a higher energy barrier than that of the H–In hydride because of the higher stability of the H–M bond than the H–In bond. Moreover, the hydride in H–M is more favorable for the formation of the monodentate HCOO* (mono-HCOO*), while the hydride in H–In is more favorable for the formation of the bidentate HCOO* (bi-HCOO*), consistent with hydrogenation of the bi-HCOO* to H_2_COO* from previous studies.^12^ Therefore, only the hydrogenation of the 
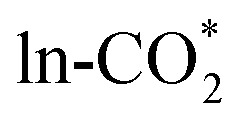
 with the H–In was studied in this work, as shown in Fig. 4(a). The energy barrier of CO_2_ hydrogenation ranges from 0.15 to 0.48 eV, indicating that this process is easy to occur. The high stability of the HCOO* is indicated by the very negative adsorption energy ranging from −4.35 to −3.19 eV. In addition, only for the Ru, Rh, Ir and Os doped surfaces, the energy barrier of this process is lower than that on the pure In_2_O_3_ surface of 0.36 eV. Low energy barriers of <0.2 eV were previously reported for Ir–In_2_O_3_ by Huang *et al.*^29^ and Chen *et al.*,^34^ and our results indicate the potential of the Os/In_2_O_3_ SAC for CO_2_ hydrogenation.

As shown in Fig. S8,† in the initial structure of the RWGS route, the bt-CO_2_ is co-adsorbed with a proton at the O_c_ site followed by the protonation of the bt-CO_2_. Unfortunately, the COOH* adsorbate is unstable on all these surfaces except for the Ag, Au, Cu, Ni and Pt doped surfaces, leading to its direct dissociation into adsorbed CO and hydroxyl group. Fig. 4(b) shows the potential energy surface for the protonation of CO_2_ to form CO on the Ag, Au, Cu, Ni and Pt doped In_2_O_3_ surfaces. The energy barrier of the protonation step ranges from 1.73 to 3.16 eV, so it is slower than the CO_2_ hydrogenation to the HCOO* intermediate in terms of both thermodynamics and kinetics. The energy barrier for further dissociation of the COOH* to form CO (TS2) ranges from 0.00 to 1.11 eV, and COOH* should readily occur on these surfaces except for the Au doped surface. Comparing the energy barriers of these two elementary steps in the RWGS route, CO_2_ protonation is the rate determining step (RDS), consistent with previous predictions.^37^ In addition, there is a decrease in the energy barrier of TS1 and an increase in the energy barrier of TS2 with the increase in the oxygen vacancy formation energy, indicating that a higher oxygen vacancy formation energy benefits CO_2_ protonation but not COOH dissociation, so an intermediate value for the oxygen vacancy formation energy should be preferable for the RWGS reaction *via* the COOH route.

**Fig. 5:**
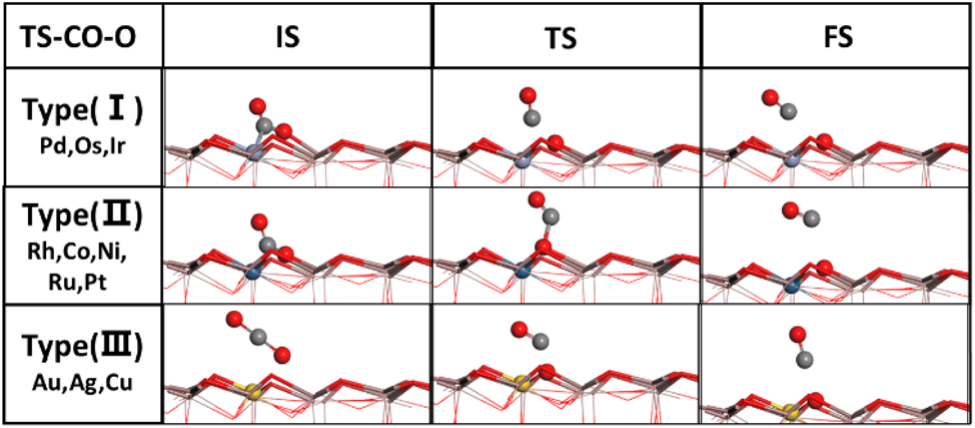
Typical initial, transition and final state (IS, TS and FS) structures of the three types of CO_2_ dissociation (I: 
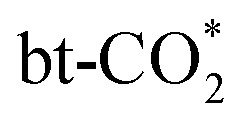
 dissociation to form surface adsorbed CO*, II: 
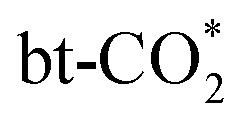
 dissociation to form gas phase CO, III: 
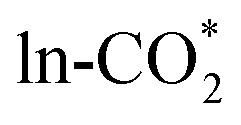
 dissociation to form gas phase CO).

The potential energy surface of CO_2_ direct dissociation is shown in Fig. 4(c), where the O* generated by CO_2_ dissociation fills the oxygen vacancy. The energy barrier ranges from 1.05 to 3.06 eV, also significantly higher than that of CO_2_ hydrogenation to the HCOO* intermediate on all doped surfaces but lower than that of CO_2_ protonation to the COOH* intermediate on the Cu and Ni doped surfaces. In addition, Fig. 4(d) shows the comparison of these energy barriers for a more intuitive display. Based on the transition state structures of CO_2_ direct dissociation, there are three types as shown in Fig. 5. Type (I) includes Pd, Os and Ir surfaces, where the transition state actually involves the breaking of both the C–O and C–M bonds in the bt-CO_2_ as CO does not adsorb on the surface. Type (II) includes Rh, Co, Ni, Ru and Pt doped surfaces, where the transition state involves only the breaking of the C–O bond as the bt-CO_2_ dissociates from the opposite configuration of type (I) to form the physisorbed CO on surface. Type (III) includes the pristine and Ag, Au, Cu doped surfaces, where the 
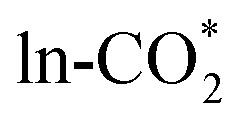
 is the initial state in CO_2_ dissociation and the transition state involves the breaking of only the C–O bond. The energy barriers of CO_2_ direct dissociation on the Co, Ru, Rh and Os doped surfaces are lower than that of the pristine surface. Furthermore, the energy barrier of the Ru/In_2_O_3_ surface is the lowest (1.05 eV) among all the studied surfaces, and the energy barrier of CO_2_ hydrogenation to the HCOO* intermediate on this surface is also very low (0.24 eV), thus Ru and Os single atom doped In_2_O_3_ catalysts may be excellent SACs for the CO_2_ hydrogenation to methanol reaction. In contrast, the energy barrier of CO_2_ direct dissociation on the Fe/In_2_O_3_ surface is very high (3.06 eV), and that of CO_2_ hydrogenation to the HCOO* intermediate is also quite high (1.41 eV), so this surface can be expected to be quite inactive to the CO_2_ hydrogenation reaction.

### 3.5 Scaling relations of CO_2_ hydrogenation and dissociation on defect surfaces

**Fig. 6:**
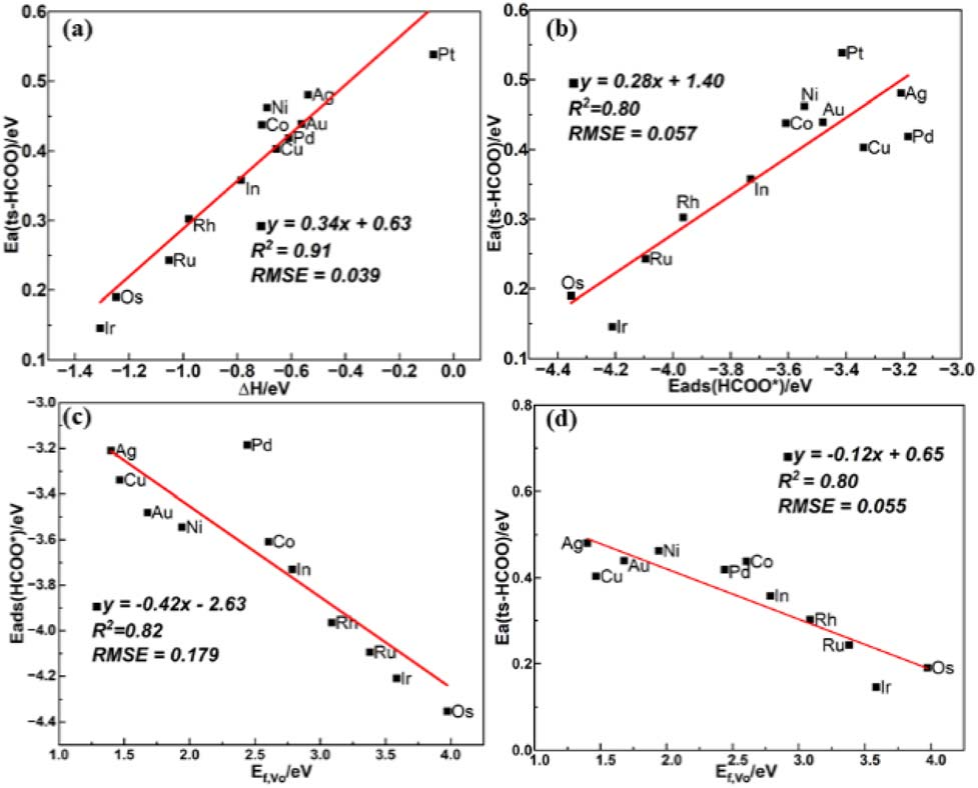
(a) BEP and (b) TSS relations of 
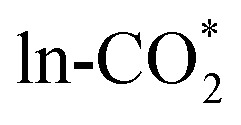
 hydrogenation to HCOO*, scaling relations between *E*_f,V_O__ and (c) *E*_ads_(HCOO*) and (d) the energy barrier of 
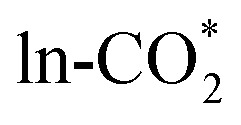
 hydrogenation to HCOO*.

For 
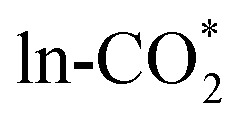
 hydrogenation to HCOO*, the BEP relationship is poor in the work of Chen *et al.*^34^ possibly due to the differences in the transition state structures. However, in this work, obvious BEP relation of this reaction and TSS relation between the energy barrier and the adsorption energy of the final state are found in Fig. 6(a) and (b), but the linear relation between the energy barrier and the adsorption energy of the initial state cannot be established, as the adsorption energy of physiosorbed CO_2_ in the initial state is approximately 0 eV, and the adsorption energy of H on the In_e_ site is similar for all the studied surfaces. From the expanded scaling relations in Fig. 6(c) and (d), both the adsorption energy of the HCOO* and the energy barrier of 
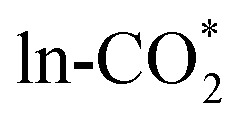
 hydrogenation to HCOO* scales linearly with the oxygen vacancy formation energy. Therefore, *E*_f,V_O__ can be used as a good descriptor for 
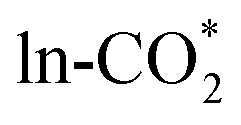
 hydrogenation to HCOO*, where a higher oxygen vacancy formation energy enhances the stability of the HCOO* and also reduces the energy barrier of 
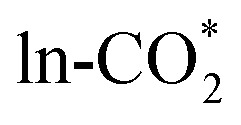
 hydrogenation, as shown in Fig. 6(d). This illustrates the crucial role of the oxygen vacancy formation energy in the CO_2_ hydrogenation reaction, which can be regulated by single metal atom doping.

For the bt-CO_2_ adsorption state, the linear correlation between the oxygen vacancy formation energy and the CO_2_ adsorption energy is not strong (*R*^2^ = 0.74) as shown in Fig. S9(a),† as previously noted by Ye *et al.*^12^ From the differential charge density analysis shown in Fig. S6,† there is an obvious charge transfer (Δ*q*) between CO_2_ and the surface ranging from 0.42 to 0.96*e*. The charge difference is concentrated in the metal single atom dopant and the surrounding atoms, and the linear correlation between Δ*q* and the CO_2_ adsorption energy is very poor as shown in Fig. S9(b),† so is the formation energy of the V_O_. However, as shown in Fig. S9(c),† the Os 5d and C 2p states are strongly hybridized on the defect Os/In_2_O_3_ surface, so here we propose a binary descriptor consisting of the d-band center of the single metal atom on the defect surface and the oxygen vacancy formation energy to correlate the adsorption energy of the 
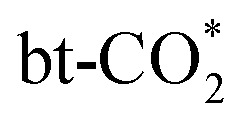
. As shown in Fig. S9(d),† a good linear relationship (*R*^2^ = 0.87) is found, indicating a synergy of the oxygen vacancy and the single metal atom sites on CO_2_ adsorption.

**Fig. 7:**
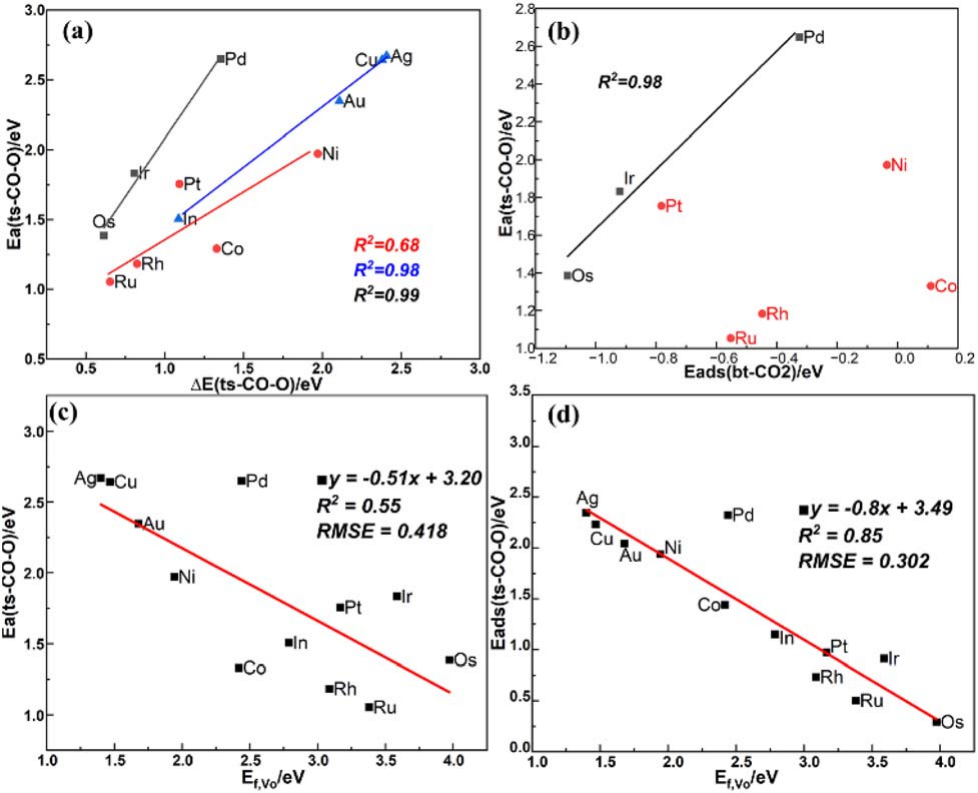
(a) BEP relation of CO_2_ dissociation, (b) TSS relation between *E*_ads_(btCO_2_) and energy barrier of ts-CO-O with type (I) (black) and type (II) (red), scaling relation between (c) *E*_f,V_O__ and the energy barrier, (d) *E*_f,V_O__ and the energy of the transition state.

The BEP relation for CO_2_ direct dissociation on all doped surfaces is not strong, but after dividing the transition states into the three types (I, II, III) as mentioned in Section 3.4, the BEP relations are much improved with the *R*^2^ values of 0.99, 0.68 and 0.98 as shown in Fig. 7(a), suggesting there are significant difference among the transition state structures of the different types, which affect the BEP relation on the single metal atom doped In_2_O_3_ surfaces. Due to the weak CO adsorption in the final state with an essentially zero adsorption energy, there is a good TSS relation between the adsorption energy of the initial state and the energy barrier as shown in Fig. 7(b). The energy barrier of CO_2_ direct dissociation decreases as the CO_2_ adsorption becomes stronger, but this trend is not obvious for type (II). The energy barrier of type (I) also scales linearly with the oxygen vacancy formation energy, indicating the crucial role of the oxygen vacancy in 
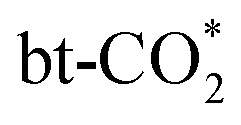
 dissociation. Although the linear relation between the oxygen vacancy formation energy (*E*_f,V_O__) and the energy barrier for CO_2_ direct dissociation is poor as shown in Fig. 7(c), the energy of the transition state can be linearly correlated with *E*_f,V_O__ with a *R*^2^ value of 0.85 as shown in Fig. 7(d), indicating that *E*_f,V_O__ can be used as a descriptor for CO_2_ direct dissociation.

For the RWGS reaction *via* the COOH* pathway, the BEP and TSS relations are both poor due to the significant differences in the transition state structures of 
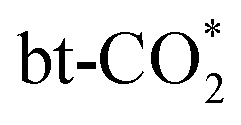
 protonation. Nonetheless, as shown in Fig. S10(a),† the energy barrier of 
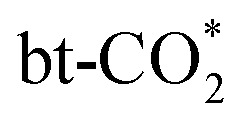
 protonation scales linearly with the 
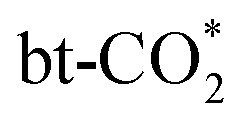
 adsorption energy when Cu/In_2_O_3_ is excluded, consistent with a previous study.^34^ Moreover, a good BEP relation can only be established for COOH* dissociation to CO* and OH* when excluding Ni and Pt doped In_2_O_3_ surfaces as the transition state of COOH* dissociation is difficult to locate as shown in Fig. S10(b).†

In addition, Fig. 4(d) shows that CO_2_ hydrogenation to the HCOO* is easier to occur than the CO_2_ dissociation *via* the COOH* and its direct dissociation. However, several recent studies suggest that In_2_O_3_ catalysts with Ni and Pt single atom dopants on the surface lead to a high CO selectivity and a low methanol selectivity. As shown in Fig. 6(d) and 7(c), the slopes of the linear relations between the energy barriers of CO_2_ hydrogenation to HCOO* and direct dissociation of CO_2_ and the oxygen vacancy formation energy are −0.12 and −0.51, respectively, indicating that CO_2_ dissociation are more sensitive to the oxygen vacancy formation energy than its hydrogenation to the HCOO*. Based on the calculated energy barriers, the predicted order of the catalytic performance of the 12 single metal atom-doped In_2_O_3_ catalysts is shown in Table S10.† For the perfect surfaces, Au/In_2_O_3_ exhibits the highest reducibility by H_2_, leading to the formation of oxygen vacancies. For the surfaces with oxygen defects, Os/In_2_O_3_ shows the optimal performance for H_2_ dissociation, Ir/In_2_O_3_ exhibits the highest activity for CO_2_ hydrogenation to HCOO*, and Ru/In_2_O_3_ demonstrates the superior activity for CO_2_ dissociation to CO. Thus, Ru/In_2_O_3_, Ir/In_2_O_3_, and Os/In_2_O_3_ may be expected to have high catalytic activities for CO_2_ hydrogenation.

### 3.6 Discussion

Previously, there have already been some scaling relations for CO_2_ conversion on oxide-supported single atom catalysts. On single metal doped t-ZrO_2_(101), Cheula *et al.*^53^ have derived linear scaling relations between the formation energy of the transition state and the co-adsorption energy of two H adatoms at the M and O sites in the HCOO* formation step with a *R*^2^ value of 0.98. As a similar scaling relation was not found in this work, we replaced the co-adsorption energy of H adatoms with the adsorption energy of HCOO* as mentioned in Section 3.5. In addition, they found the formation energy of the transition state for H_2_ dissociation was also linearly correlated with the co-adsorption energy of two H adatoms at the M and O site, whereas we found the adsorption energy of H at the O site to be sufficient.

Moreover, increasing the coverage of 
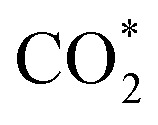
 on the catalyst surface should promote its reaction, so a strong CO_2_ adsorption will usually benefit the CO_2_ conversion rate.^54^ In a previous study,^55^ comparison of the linear and bent CO_2_ adsorption energies over the TiO_2_ with metal adatoms (M/TiO_2_) surfaces shows that adsorptions of the linear and bent CO_2_ over the TiO_2_ surface are much weaker than those on the surfaces with metal adatoms, indicating the likely important role of the metal atom dopant in CO_2_ adsorption and reduction. Our calculations show that ln-CO_2_ adsorption is strengthened on most metal doped surfaces except for Ag, Au, Pt, Os, Pd, as shown in Table S8.† In addition, CO_2_ dissociation is more favorable on early transition metal doped surfaces such as Hf and W rather than late transition metals such as Cu and Pt as the CO_2_ dissociation energy becomes more negative from the early to late transition metal element in the same period, similar results are also found in this work as shown in Fig. S10(c).† An approximately linear correlation between the CO_2_ dissociation energy and the bt-CO_2_ adsorption energy is found with a *R*^2^ value of 0.78,^55^ so capacity for CO_2_ activation is positively correlated with that of CO_2_ reduction, and there is a similar but poorer linear correlation between the CO_2_ dissociation energy and the bt-CO_2_ adsorption energy as shown in Fig. S10(d)† with a *R*^2^ value of 0.57. In order to understand the linear scaling relations between CO_2_ adsorption strength and its dissociation barrier, correlations between the adsorption energy, dissociation barrier, and excess charge on the surface of TiO_2_, Al_2_O_3_, and CeO_2_ with single metal atoms (M) are explored.^56^ The linear relation between the energy barrier of CO_2_ direct dissociation and the Hirshfeld charge suggests that a more negative charge on the single atom correlates with a lower energy barrier, but a similar correlation cannot be established from our results, as shown in Fig. S9(b).† Systems with stronger CO_2_ adsorption also have a lower energy barrier for CO_2_ direct dissociation from their linear scaling relation with a *R*^2^ value of 0.73 for M/Al_2_O_3_, consistent with our results as shown in Fig. 7(b). In addition, the bt-CO_2_ adsorption structure is not stable on Ag/CeO_2_ or Ag/Al_2_O_3_, indicating a weaker adsorption and a less activated CO_2_ adsorbate. They found CO_2_ adsorption on Cu/Al_2_O_3_ and Ag/Al_2_O_3_ surfaces to be weaker than the Rh/Al_2_O_3_ surface, which is consistent with our results for the corresponding M/In_2_O_3_ surfaces (CO_2_ adsorption energies are −0.32 eV, −0.31 eV, and −0.47 eV for Cu/In_2_O_3_, Ag/In_2_O_3_ and Rh/In_2_O_3_, respectively).

## 4. Conclusions

In this study, we investigated the mechanism of H_2_ and CO_2_ activations on single metal atom doped In_2_O_3_ catalyst to reveal the effective catalyst descriptors influencing CO_2_ and H_2_ activation through scaling relations. For the dissociative adsorption of H_2_ on the perfect doped surfaces, the formation of In–O pairs through heterolytic dissociation remains feasible and good BEP and TSS relations are found. The adsorption energy of the H adatom at the O site serves as an effective descriptor for the energy barrier of H_2_ dissociation, which can be further described in terms of the d band center of the single metal dopant and the p band center of the O site. Our calculations further show that single atom catalysts formed by Au, Ag and Cu doping can readily induce the formation of adjacent oxygen vacancies. Secondly, we conducted investigations into the activation of H_2_ and CO_2_ on surfaces with oxygen vacancy and identified Os, Ru and Ir/In_2_O_3_ as promising single-atom catalysts (SACs) for CO_2_ hydrogenation. Thirdly, we found that CO_2_ hydrogenation is significantly easier than protonation and is greatly influenced by the formation energy of oxygen vacancies. The formation energy of oxygen vacancies, acting as a descriptor, negatively scale linearly with energy barriers of both CO_2_ hydrogenation to HCOO and dissociation to CO and the effect on the latter is greater.

## Data availability

The data supporting this article have been included as part of the ESI.†

## Author contributions

Yuanjie Bao: investigation, data curation, writing – original draft, visualization; Ziqi Tang: investigation, data curation, visualization; Yuchen Wang: methodology, validation, visualization; Shenggang Li: conceptualization, validation, resources, supervision, project administration, funding acquisition.

## Conflicts of interest

The authors declare that they have no known competing financial interests or personal relationships that could have appeared to influence the work reported in this paper.

## Supplementary Material

RA-015-D4RA09111F-s001
