# Supplementary material for: Scaling relations of CO2 hydrogenation and dissociation on single metal atom doped In2O3 catalysts with promoted oxygen vacancy sites
Source: RSC Adv. 2025 Mar 12;15(10):7832–42. doi: 10.1039/d4ra09111f (PMC11897883; doi:10.1039/d4ra09111f)
Supplement: RA-015-D4RA09111F-s001 [file RA-015-D4RA09111F-s001.pdf]

Supplementary Information (SI) for RSC Advances.
This journal is © The Royal Society of Chemistry 2025

Electronic Supplementary Information for

Scaling Relations of CO_2 Hydrogenation and Dissociation on Single Metal Atom Doped In_2O_3 Catalysts with Promoted Oxygen Vacancies

Yuanjie Bao[a,b], Ziqi Tang[a,c], Yuchen Wang[a,c] and Shenggang Li [a,b,c,d]*

[a]*CAS Key Laboratory of Low−Carbon Conversion Science and Engineering, Shanghai Advanced Research Institute, Chinese Academy of Sciences, Shanghai 201203, P. R. China*

[b]*University of Chinese Academy of Sciences, Beijing 100049, P. R. China*

[c]*School of Physical Science and Technology, ShanghaiTech University, Shanghai 201210, P. R. China*

[d]*State Key Laboratory of Low Carbon Catalysis and Carbon Dioxide Utilization, Shanghai Advanced Research Institute, Chinese Academy of Sciences, Shanghai 201210, China*

**Corresponding author. Tel: +86–021–20350994.*

E-mail: lisg@sari.ac.cn (S. Li)

Linear regression analysis: We solve the regression coefficients by minimizing the sum of squared errors using the least squares method [1]. The correlation coefficient R^2 is calculated by: $R^2 = 1 - \frac{\sum_{i=1}^{n}(y_i - \hat{y}_i)^2}{\sum_{i=1}^{n}(y_i - \bar{y})^2}$, the Root Mean Square Error (RMSE) is calculated by $RMSE = \sqrt{(\Sigma(y_i - \hat{y}_i)^2/n)}$. The linear scaling relations in this work are expressed with the 95% confidence interval for slopes and intercepts as the significance level (α) is set at 0.05.

[1] Ryan, T. P. Modern Regression Methods. New York, John Wiley & Sons (1997).

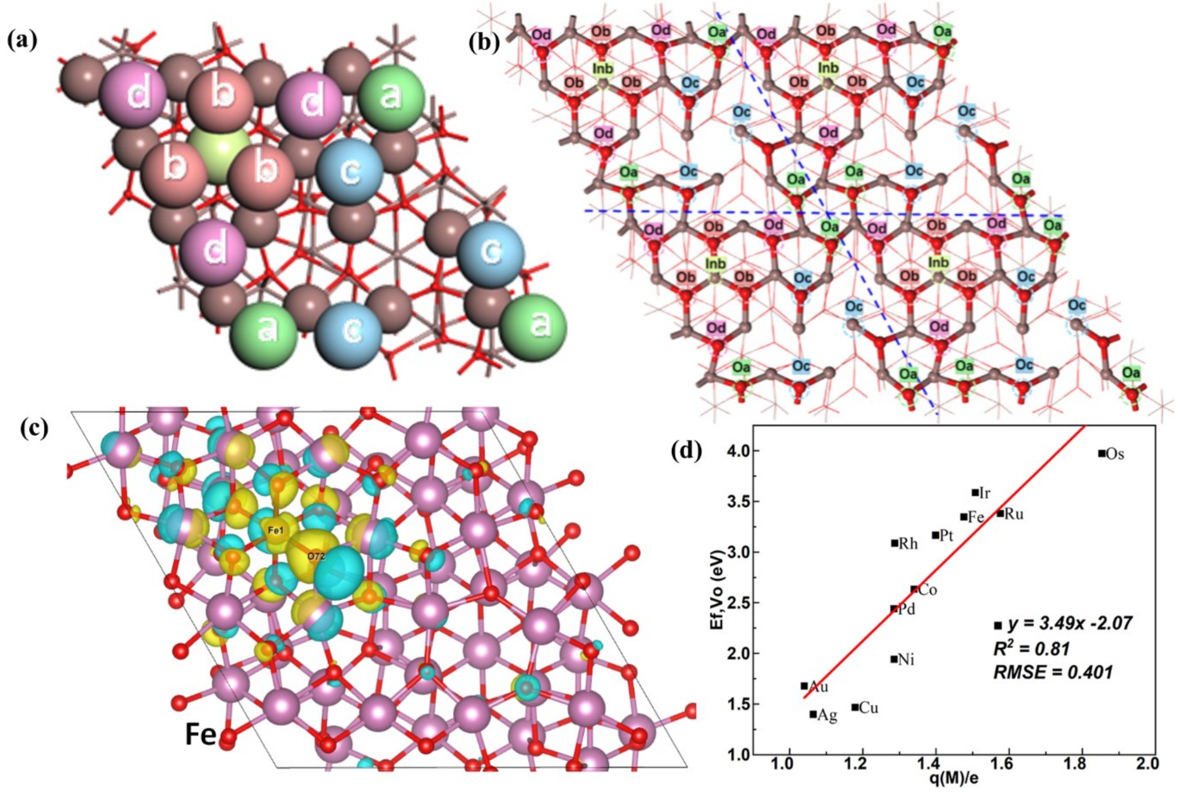

Figure S1. (a) Different O sites on the $In_2O_3(111)$ surface with a single atom doped at the In_b site, (b)A 2×2 arrangement of the primitive unit cell with labelled O atoms (a ~ d) relatively to the In_b atom, (c) The differential charge density of Fe/In_2O_3 surfaces, in which light blue and yellow regions indicate charge accumulation and depletion, respectively, (d) Scaling relation between q(M) and $E_{f,Vo}$.

Table S1. Bader charge analysis for single atoms and oxygen atoms on all doped surfaces .

Surface/site number	Charge	Oa			Ob			Oc			Od		
	/|e|	10	12	4	5	6	3	9	11	7	3	8	1
Fe	1.48	-1.18	-1.18	-1.18	-0.98	-1.01	-0.98	-1.15	-1.16	-1.15	-1.17	-1.17	-1.16
Co	1.34	-1.18	-1.18	-1.18	-0.99	-1.00	-0.99	-1.15	-1.16	-1.15	-1.17	-1.17	-1.16
Ni	1.29	-1.18	-1.18	-1.18	-0.98	-0.98	-1.00	-1.15	-1.16	-1.15	-1.17	-1.17	-1.17
Cu	1.18	-1.18	-1.18	-1.18	-0.98	-0.98	-1.10	-1.15	-1.16	-1.15	-1.16	-1.17	-1.17
Ru	1.58	-1.18	-1.18	-1.19	-0.96	-0.97	-0.97	-1.15	-1.16	-1.15	-1.17	-1.17	-1.17
Rh	1.29	-1.18	-1.18	-1.18	-0.98	-0.99	-0.99	-1.15	-1.16	-1.15	-1.17	-1.17	-1.17
Pd	1.28	-1.18	-1.18	-1.19	-0.95	-0.95	-0.96	-1.15	-1.16	-1.16	-1.17	-1.17	-1.17
Ag	1.06	-1.18	-1.18	-1.18	-0.96	-0.96	-1.09	-1.14	-1.15	-1.14	-1.16	-1.16	-1.17
Os	1.85	-1.18	-1.18	-1.18	-0.98	-0.98	-0.99	-1.15	-1.16	-1.16	-1.16	-1.17	-1.16
Ir	1.51	-1.18	-1.18	-1.18	-0.98	-0.99	-0.99	-1.15	-1.16	-1.15	-1.17	-1.17	-1.17
Pt	1.40	-1.18	-1.18	-1.18	-0.97	-0.97	-0.98	-1.15	-1.16	-1.16	-1.17	-1.17	-1.17
Au	1.04	-1.18	-1.18	-1.18	-0.97	-0.96	-1.08	-1.14	-1.16	-1.15	-1.15	-1.17	-1.17
In	1.89	-1.18	-1.18	-1.18	-1.15	-1.15	-1.15	-1.14	-1.15	-1.15	-1.16	-1.16	-1.17

Table S2. Energy barrier (Ea), reaction energy (ΔH) and adsorption energy of initial state (IS) and final state (FS) of H_2 heterolysis, and Eads(H@In) on all doped perfect surfaces.

Surface	Ea/eV	ΔH/eV	IS/eV	FS/eV	Eads(H@In)/eV
Fe	0.74	-0.75	0.27	-0.48	/
Co	0.73	-0.61	-0.04	-0.65	0.87
Ni	0.81	-0.62	-0.09	-0.71	/
Cu	0.67	-0.73	-0.06	-0.79	/
Ru	0.80	-0.37	-0.07	-0.44	0.83
Rh	0.77	-0.48	-0.07	-0.56	0.85
Pd	0.80	-0.30	-0.07	-0.38	/
Ag	0.58	-0.71	-0.05	-0.77	/
Os	0.81	-0.31	-0.07	-0.38	/
Ir	0.82	-0.36	-0.07	-0.43	/
Pt	0.84	-0.27	-0.07	-0.34	0.85
Au	0.56	-0.69	-0.05	-0.74	/
In	0.88	-0.41	-0.05	-0.46	/
Ag-homolytic	/	/	-0.05	-3.7	

Table S3. Energy barrier (Ea), reaction energy (ΔH) and adsorption energy of initial state (IS) and final state (FS) of H_2O formation on all doped perfect surfaces.

Surface	Ea/eV	ΔH/eV	IS/eV	FS/eV
Fe	1.92	-0.87	-0.48	-1.19
Co	1.06	-0.61	-0.65	-0.98
Ni	1.11	-1.24	-0.71	-0.97
Cu	0.87	-1.57	-0.79	-0.91
Ru	1.02	-0.11	-0.44	-1.01
Rh	1.06	-1.97	-0.56	-2.69
Pd	1.22	-0.94	-0.38	-0.84
Ag	0.80	-1.47	-0.77	-0.71
Os	1.16	0.51	-0.38	-0.92
Ir	1.11	0.04	-0.43	-1.06
Pt	1.35	-0.25	-0.34	-0.84
Au	0.73	-1.15	-0.74	-0.65
In	0.96	-0.51	-0.46	-0.84
Ag- homolytic	1.49	2.99	-3.70	-0.71

Table S4. Bond lengths of In-H, O-H, H-H of transition state (TS) and final state (FS) of H_2 heterolysis on all doped perfect surfaces.

Surface	TS/Å			FS/Å			Difference/Å		
	In-H	O-H	H-H	In-H	O-H	H-H	In-H	O-H	H-H
Fe	2.14	1.28	0.97	1.78	0.97	3.01	0.36	0.31	2.04
Co	2.16	1.27	0.98	1.78	0.97	3.14	0.39	0.30	2.16
Ni	2.15	1.28	0.98	1.79	0.97	3.14	0.36	0.31	2.16
Cu	2.23	1.24	0.98	1.80	0.97	3.01	0.42	0.27	2.03
Ru	1.98	1.06	1.26	1.78	0.97	3.05	0.21	0.09	1.78
Rh	2.14	1.27	0.99	1.78	0.97	3.14	0.36	0.30	2.15
Pd	2.13	1.24	1.00	1.77	0.97	3.11	0.36	0.26	2.11
Ag	2.37	1.30	0.96	1.81	0.97	2.99	0.56	0.34	2.03
Os	2.09	1.30	0.95	1.77	0.97	3.00	0.32	0.34	2.04
Ir	2.11	1.27	0.99	1.78	0.97	3.09	0.33	0.30	2.11
Pt	2.09	1.26	1.00	1.78	0.97	3.11	0.32	0.28	2.11
Au	2.45	1.29	0.97	1.81	0.97	2.92	0.64	0.32	1.96
In	2.14	1.25	0.96	1.78	0.98	3.00	0.36	0.27	2.04

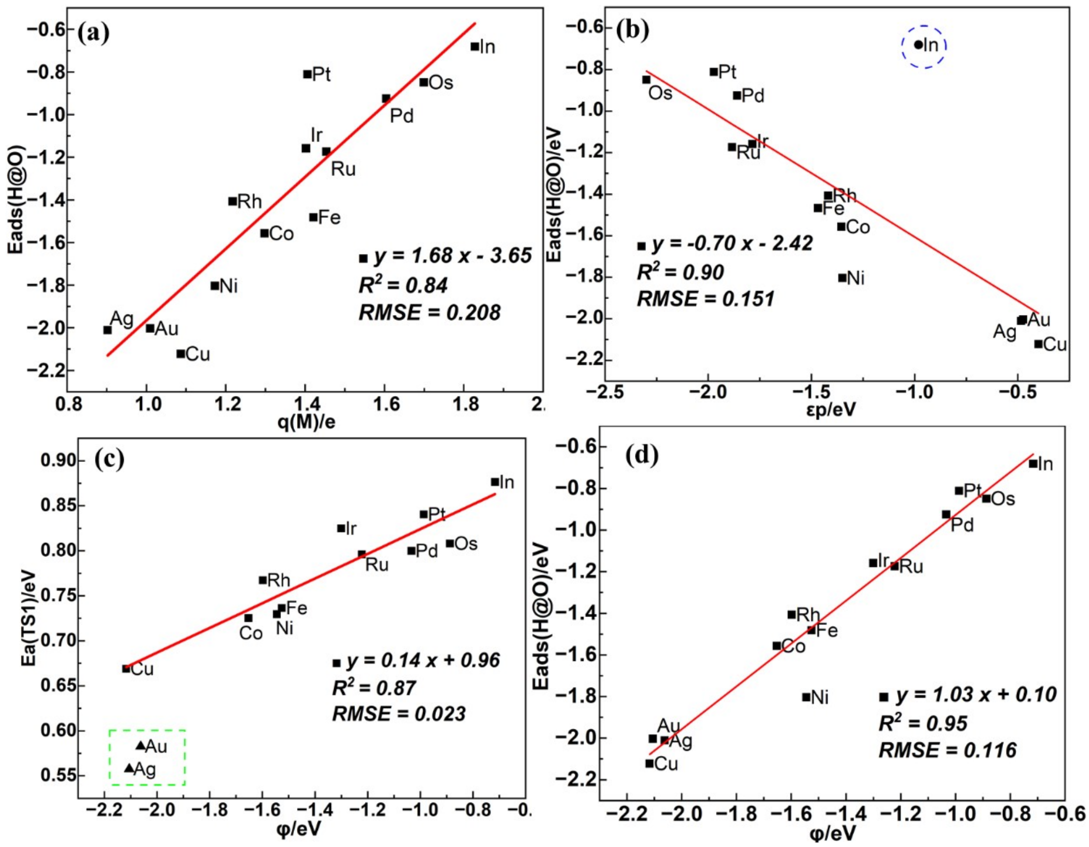

Figure S2. Scaling relation between (a) Bader charge of single metal atom on perfect surface q(M) and Eads(H@O), (b) ε_p and Eads(H@O), (c)φ and Ea (TS1), (d) φ and Eads(H@O).

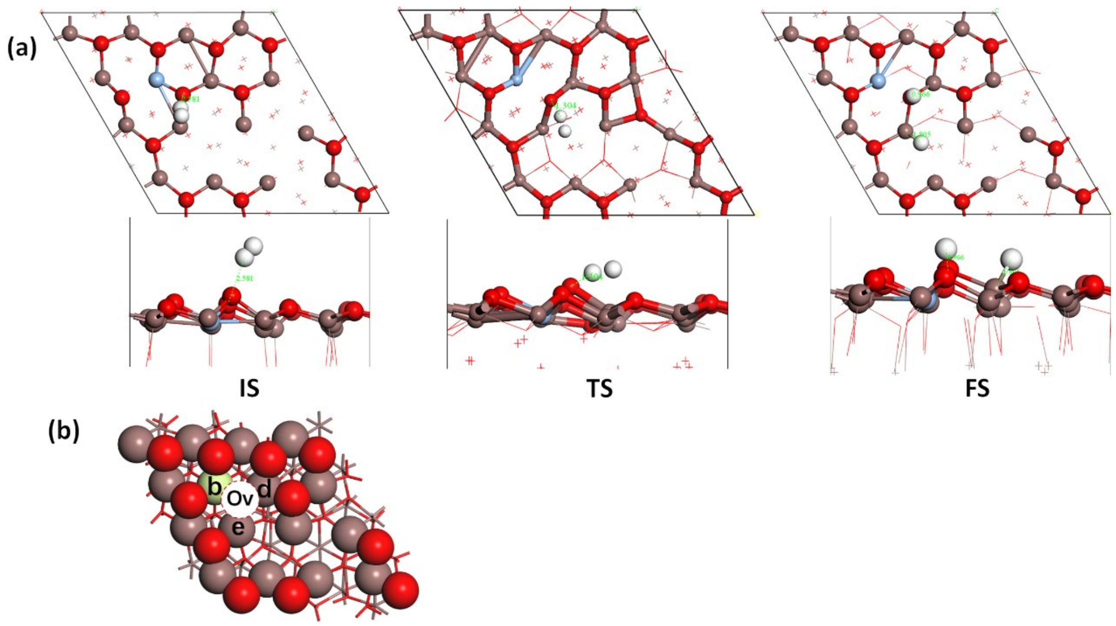

Figure S3. (a)The initial, transition and final state of H_2 heterolysis on Ag/In_2O_3, the reaction sites are all the same on other doped surfaces. (b) the In_2O_3(111) surface doped with a single metal atom at the In_b site and an oxygen vacancy.

Table S5. The adsorption energy of H adatoms at the H@In&H@O and E_{ads}(H@In) on all defect surfaces.

Surface	E(H@In&H@O)/eV	E_{ads}(H@In)/eV
Fe	-0.69	0.29
Co	-0.75	/
Ni	-0.61	0.37
Cu	-0.71	/
Ru	-0.78	/
Rh	-0.77	/
Pd	-0.79	0.36
Ag	-0.78	/
Os	-0.69	/
Ir	-0.76	/
Pt	-0.72	0.33
Au	-0.79	/
In	-0.80	/

Table S6. Energy barrier (Ea), reaction energy (ΔH) and adsorption energy of final state (FS) of H_2 dissociation by pathway (4) on partial doped defect surfaces.

Surface	Ea/eV	ΔH/eV	Eads(H@In&H@O)/eV
In	0.82	-0.74	-0.80
Pt	0.82	-0.66	-0.72
Rh	0.82	-0.71	-0.77
Ru	0.83	-0.72	-0.78
Ir	0.84	-0.70	-0.76

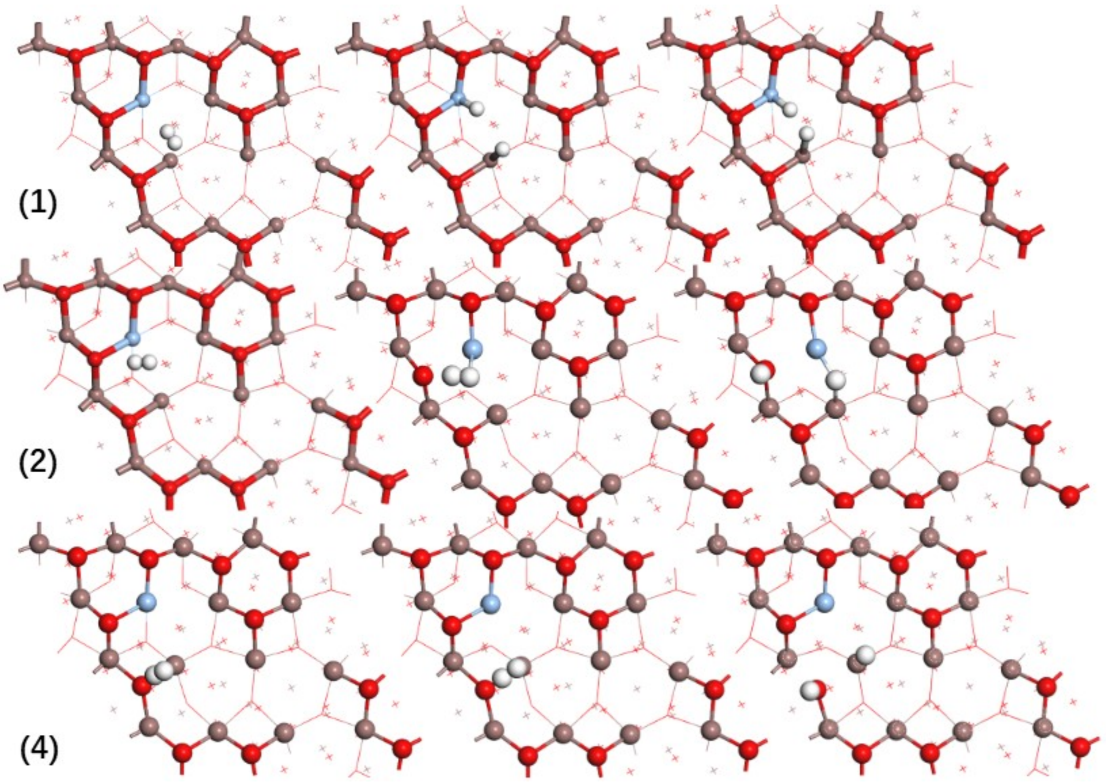

Figure S4. The initial, transition and final states of pathway (1,2 and 4) on Ag doped defect surface.

Table S7. Energy barrier (Ea), reaction energy (ΔH) and adsorption energy of initial state (IS) and final state (FS) of H_2 heterolysis by pathway (1) and (2) on all doped defect surfaces.

Surface	pathway (1)				pathway (2)			
	Ea/eV	ΔH/eV	IS/eV	FS/eV	Ea/eV	ΔH/eV	IS/eV	FS/eV
Fe	1.05	0.82	-0.02	0.79	0.28	-1.06	0.78	-0.29
Co	0.87	0.58	0.12	0.69	0.42	-1.00	0.12	-0.89
Ni	0.87	0.80	-0.03	0.78	0.48	-0.89	-0.03	-0.91
Cu	1.36	1.17	-0.08	1.09	0.64	-0.97	-0.08	-1.05
Ru	0.73	0.40	-0.06	0.34	0.00	-1.16	-0.06	-1.22
Rh	0.74	0.06	-0.06	0.00	0.03	-1.52	-0.06	-1.58
Pd	0.54	0.39	-0.06	0.33	1.02	-0.89	-0.06	-0.95
Ag	1.38	1.36	-0.07	1.29	0.48	-1.43	-0.07	-1.50
Os	0.19	-0.66	-0.05	-0.71	0.08	-1.24	-0.05	-1.29
Ir	0.44	-0.48	-0.06	-0.54	0.06	-1.74	-0.06	-1.80
Pt	0.39	-0.39	-0.06	-0.44	1.01	0.48	-0.06	0.42
Au	1.26	0.93	-0.06	0.87	0.71	-1.43	-0.06	-1.48
In	1.90	1.02	-0.07	0.95	0.90	/	-0.07	/

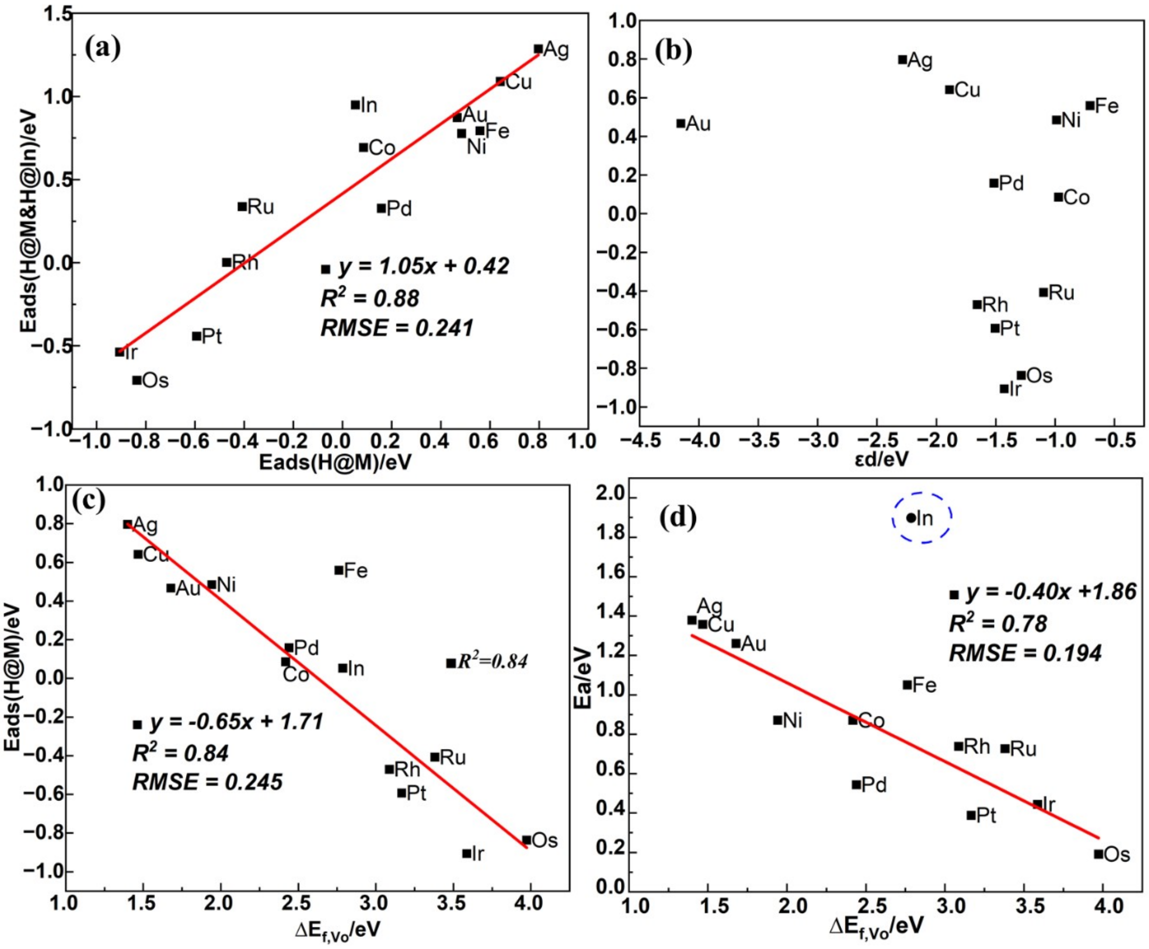

Figure S5. Scaling relation between (a) Eads(H@M) and Eads(H@M&H@In), (b) εd and Eads(H@M), (c) $\Delta E_{f,Vo}$ and Eads(H@M), (d) the energy barrier of pathway (1) and $E_{f,Vo}$.

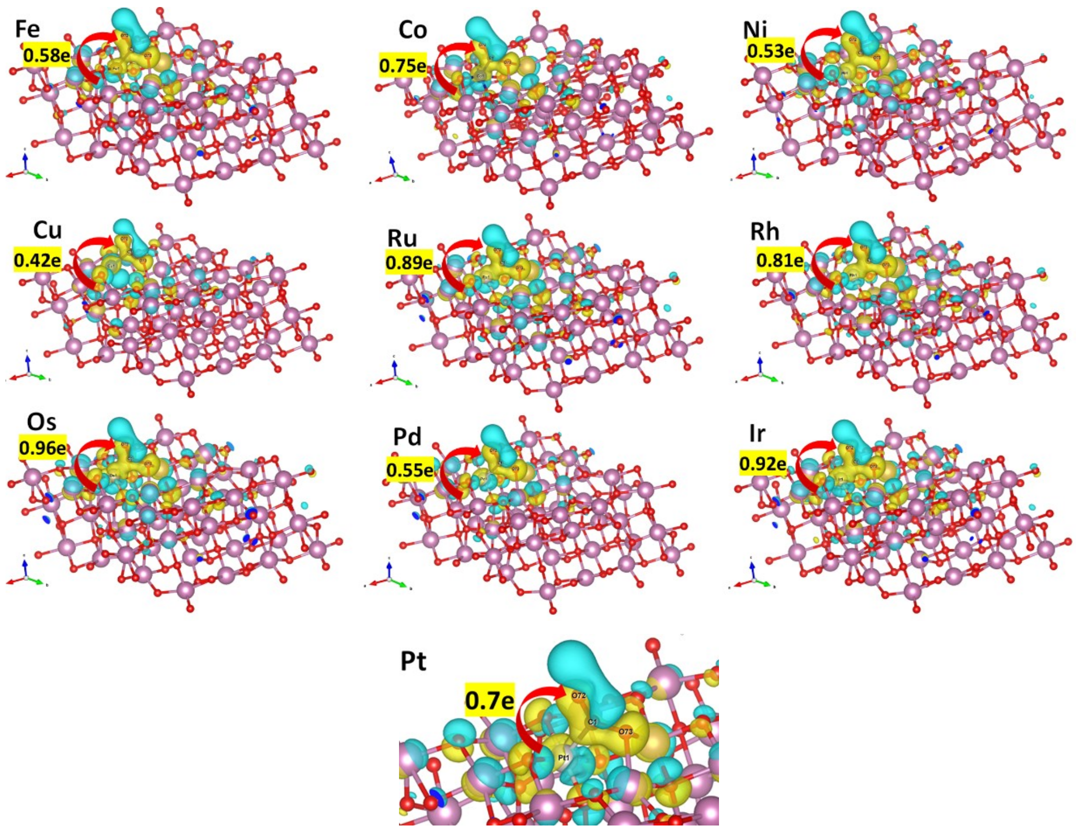

Figure S6. The differential charge density of bt-CO_2 adsorbs on doped defect surfaces. (O73 refers to O1, O72 refers to O2)

Table S8. The adsorption energy (eV) of H adatom, linear and bent CO_2 and their co-adsorption.

	H@Ine	ln-CO_2	ln-CO_2+h	bt-CO_2	bt-CO_2+h
Fe	0.07	-0.45	-0.42	0.14	-0.25
Co	0.10	-0.43	-0.10	0.11	-0.48
Ni	0.37	-0.47	-0.05	-0.03	-0.33
Cu	0.26	-0.41	0.12	-0.41	-0.58
Ru	/	-0.48	-0.24	-0.55	-0.91
Rh	0.06	-0.47	-0.19	-0.45	-0.93
Pd	0.36	-0.31	0.22	-0.32	-0.56
Ag	0.35	-0.32	0.13	-0.32	-0.53
Os	-0.14	-0.36	-0.31	-1.09	-1.34
Ir	0.02	-0.43	-0.10	-0.92	-1.32
Pt	0.40	-0.27	-0.82	-0.78	-1.05
Au	0.03	-0.31	-0.12	-0.31	-0.60
In	0.05	-0.37	-0.14	-0.36	-1.32

Table S9. Energy barriers of ln-CO_2 hydrogenation pathways by H-In and H-M hydrides on Ag, Ni, Os, Ir, Pd/In_2O_3 surfaces.

Ea(ts-HCOO)/eV		
Surface	In-H	M-H
Ag	0.48	0.52
Ni	0.46	1.30
Os	0.15	0.60
Ir	0.19	0.71
Pd	0.42	0.78

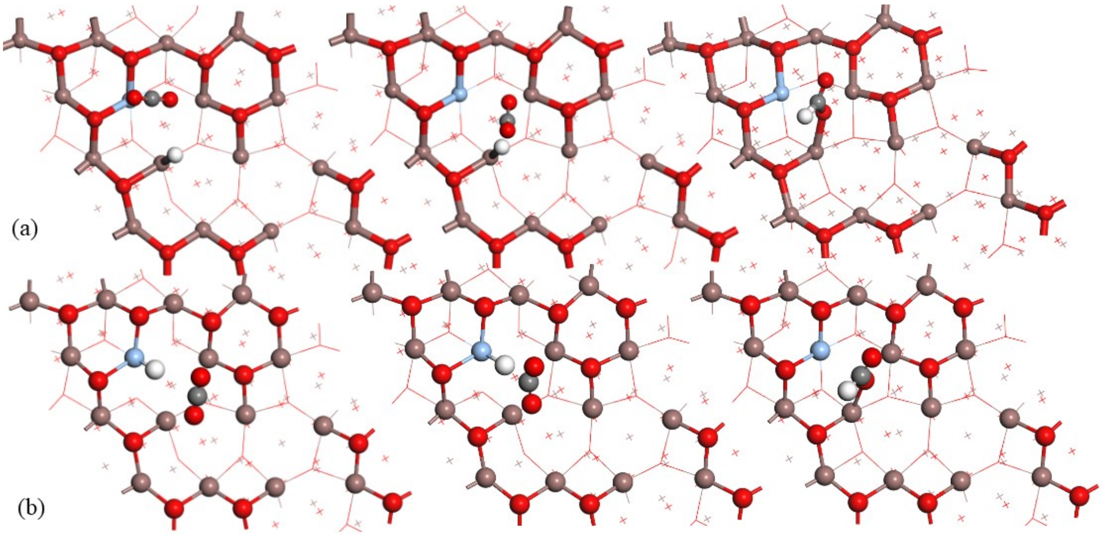

Figure S7. The IS, TS and FS structures of ln-CO_2 hydrogenation pathways by H-In (a) and H-M (b) hydrides on Ag/In_2O_3 surface.

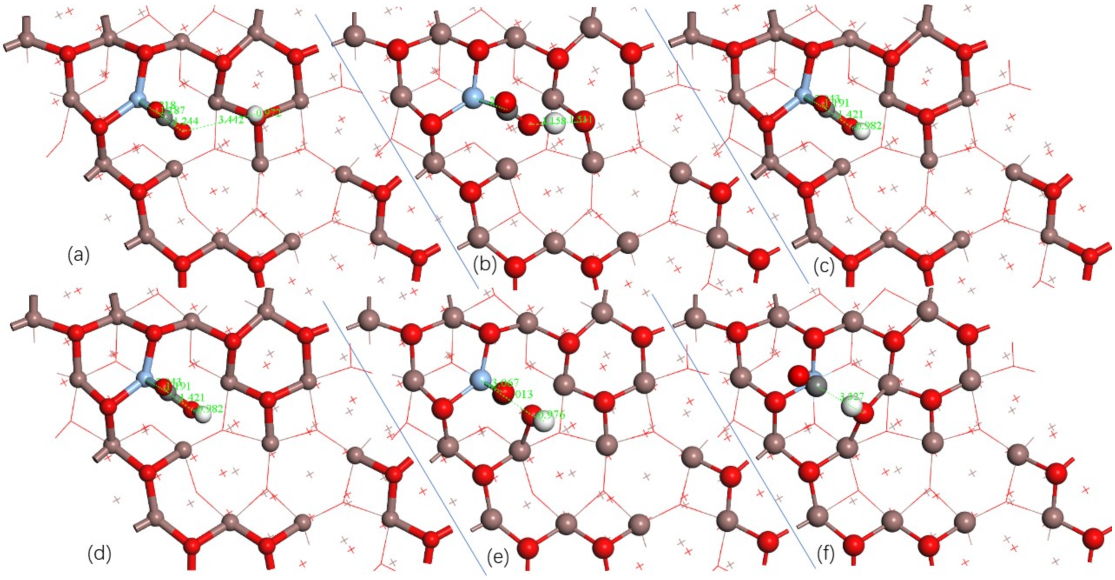

Figure S8. The IS (a), TS (b) and FS (c) structures of bt-CO_2 protonation to COOH (TS1) and IS (d), TS (e) and FS (f) structures of COOH to CO+OH (TS2) on Ag/In_2O_3 surface.

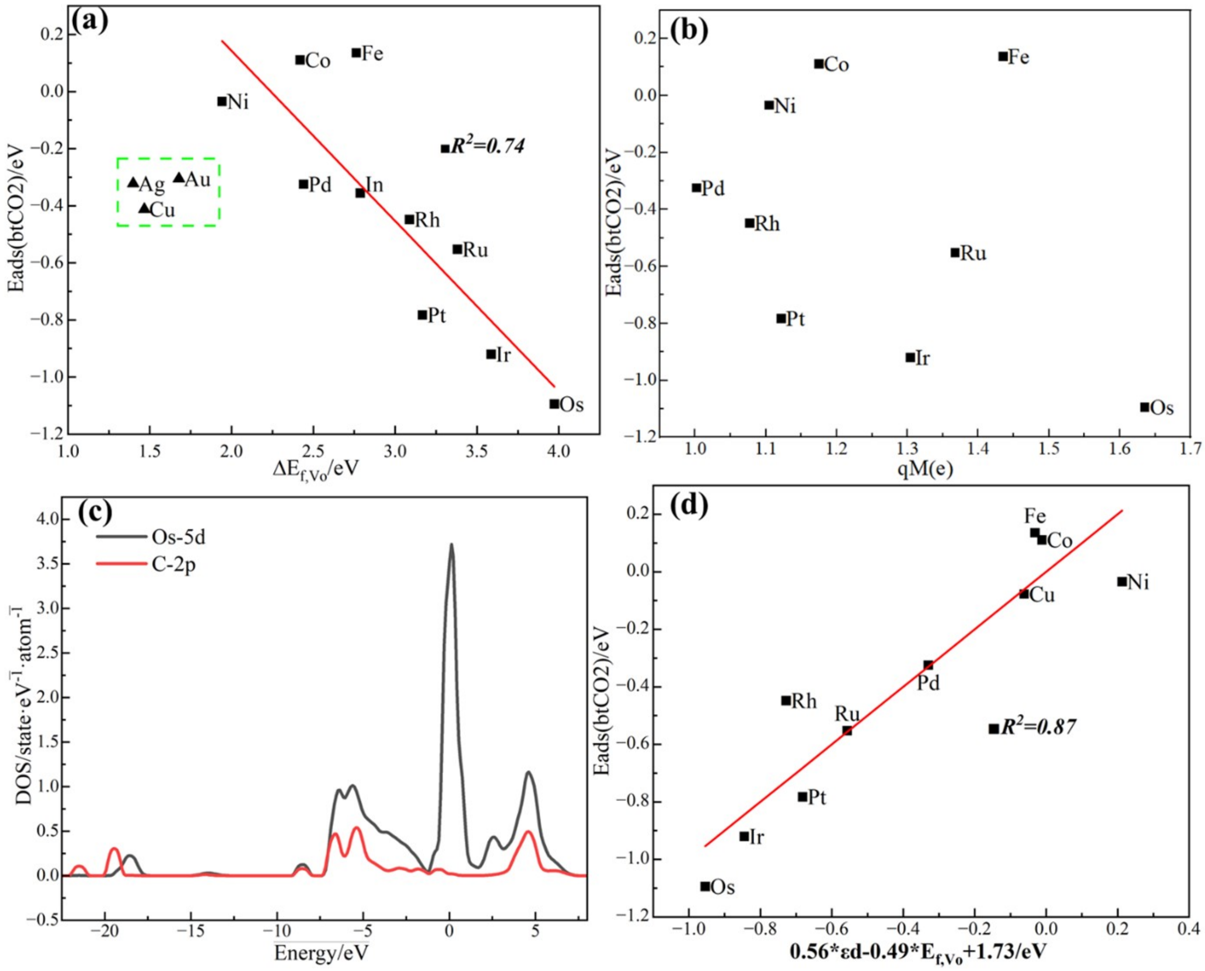

Figure S9. Scaling relations between (a) $\Delta E_{f,Vo}$ and Eads($btCO_2$), (b) qM(e) and Eads($btCO_2$); (c) Density of states projected onto the Os 5d and C 2p orbitals when bt-CO_2 absorbed on defect Os/In_2O_3 surface, (d) Scaling relations between $\Delta E_{f,Vo}$&ε_d and E_{ads}(bt-CO_2).

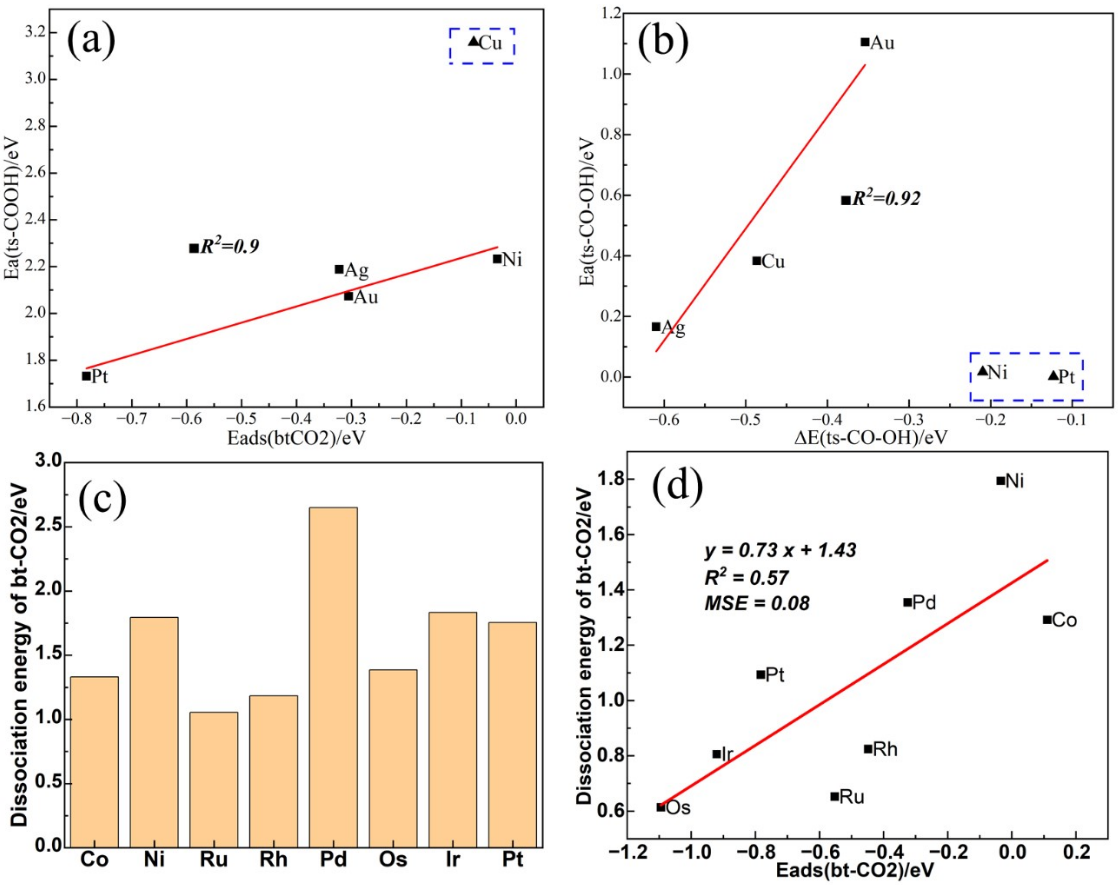

Figure S10. (a)Scaling relation between Eads(btCO_2) and energy barrier of btCO_2 protonation, (b) BEP relation of COOH dissociation to CO and OH, (c) Dissociation energy of bt-CO_2 on defective surface, (d) Scaling relations between Eads(bt-CO_2) and Dissociation energy of bt-CO_2.

Table S10. Performance order of each reaction on all 12 metal atoms doped In_2O_3 (111) surfaces from high to low.

Reaction	Performance order
H_2 heterolysis on the perfect surface	Au>Ag>Cu>Co>Fe>Rh>Ru>Pd>Os>Ni>Ir>Pt>In
Hydroxyl pronation to H_2O	Au>Ag>Cu>In>Ru>Rh>Co>Ni>Ir>Os>Pd>Pt>Fe
H_2 heterolysis on the defect surface by pathway 1	Os>Pt>Ir>Pd>Ru>Rh>Co>Ni>Fe>Au>Cu>Ag>In
H_2 heterolysis on the defect surface by pathway 2	Ru>Rh>Ir>Os>Fe>Co>Ni>Ag>Cu>Au>In>Pt>Pd
CO_2 hydrogenation to HCOO	Ir>Os>Ru>Rh>In>Cu>Pd>Co>Au>Ni>Ag>Pt>Fe
CO_2 directly dissociation to CO	Ru>Rh>Co>Os>In>Pt>Ir>Ni>Au>Cu>Pd>Ag>Fe

Table S11. Optimized factional coordinates.

The structure of the Ag/In_2O_3(111) model (structures of other catalyst models are similar)

In Ag O

1.000000000000000

12.5091307398836005 -7.2221500000000001 0.0000000000000000

0.0000000000000000 14.4443000000000001 -0.0000000000000248

0.0000000000000000 0.0000000000000000 17.9854999999999983

47 1 72

Selective dynamics

Direct

0.8515800000000000	0.4089300000000000	0.3473000000000001	T	T	T
0.5145400000000000	0.7424400000000002	0.5103100000000003	T	T	T
0.1830500000000000	0.0809500000000000	0.6840900000000002	T	T	T
0.8290999999999999	0.9356200000000000	0.3251800000000001	T	T	T
0.4948600000000000	0.2680000000000000	0.4989600000000002	T	T	T
0.1588100000000000	0.6049800000000001	0.6580000000000003	T	T	T
0.3248900000000000	0.4314100000000001	0.3251800000000001	T	T	T
0.9928500000000000	0.7640700000000001	0.4944000000000001	T	T	T
0.6667400000000000	0.1018500000000000	0.6683200000000002	T	T	T
0.8403400000000001	0.6722700000000001	0.3362400000000001	T	T	T
0.5052800000000000	0.0059399999999999	0.5051600000000002	T	T	T
0.1749300000000000	0.3327200000000000	0.6690300000000001	T	T	T
0.1065200000000000	0.6835200000000000	0.3251800000000001	T	T	T
0.7767700000000000	0.0129299999999999	0.4941100000000001	T	T	T
0.4481600000000000	0.3575600000000000	0.6808300000000003	T	T	T
0.5994800000000000	0.4426500000000000	0.3473000000000001	T	T	T
0.2664600000000000	0.7776800000000000	0.5103500000000001	T	T	T
0.9308800000000002	0.1055700000000001	0.6859000000000002	T	T	T
0.0952800000000000	0.4426500000000000	0.3473000000000001	T	T	T
0.7636200000000001	0.7725900000000001	0.5175400000000001	T	T	T
0.4392400000000000	0.1210800000000000	0.6787200000000001	T	T	T
0.0727900000000000	0.9018900000000001	0.3251800000000001	T	T	T
0.7437000000000000	0.2279500000000000	0.4948100000000002	T	T	T
0.4060400000000001	0.5611900000000001	0.6563800000000001	T	T	T
0.5657600000000000	0.6610300000000000	0.3473000000000001	T	T	T
0.2332100000000000	0.9939100000000001	0.5173900000000001	T	T	T
0.8941500000000001	0.3216100000000001	0.6796300000000002	T	T	T
0.8403400000000001	0.1680700000000000	0.3362400000000000	T	T	T
0.5018700000000000	0.5015500000000002	0.5041900000000001	T	T	T
0.1635400000000000	0.8357700000000000	0.6709900000000001	T	T	T
0.5657600000000000	0.1568300000000000	0.3473000000000002	T	T	T
0.2298700000000000	0.4953100000000000	0.5111800000000001	T	T	T
0.9040700000000000	0.8284100000000001	0.6845300000000001	T	T	T

0.1065200000000000	0.1793100000000000	0.3251800000000001	T	T	T
0.7754900000000000	0.5123900000000000	0.4991700000000002	T	T	T
0.4504800000000000	0.8524900000000000	0.6579900000000002	T	T	T
0.5770000000000000	0.9018900000000001	0.3251800000000001	T	T	T
0.2441800000000000	0.2324800000000000	0.4932200000000002	T	T	T
0.9085900000000001	0.5646300000000001	0.6719400000000001	T	T	T
0.3473800000000000	0.9131300000000000	0.3473000000000002	T	T	T
0.0150100000000000	0.2437200000000000	0.5173600000000003	T	T	T
0.6818800000000000	0.5757400000000001	0.6858100000000001	T	T	T
0.3361400000000000	0.1680700000000000	0.3362400000000000	T	T	T
0.0004100000000000	0.5036600000000001	0.5062600000000002	T	T	T
0.6770200000000000	0.8463000000000001	0.6705600000000002	T	T	T
0.3361400000000000	0.6722700000000001	0.3362400000000001	T	T	T
0.0041600000000000	0.0037300000000000	0.5055800000000003	T	T	T
0.6724800000000000	0.3392200000000001	0.6765800000000001	T	T	T
0.2541800000000000	0.7461800000000001	0.3944800000000001	T	T	T
0.9223300000000000	0.0775700000000000	0.5658100000000003	T	T	T
0.0094700000000000	0.5312000000000001	0.3799400000000001	T	T	T
0.6764000000000000	0.8603700000000001	0.5494600000000002	T	T	T
0.3452400000000000	0.1913000000000000	0.7148700000000001	T	T	T
0.9439200000000000	0.1009500000000000	0.3071900000000001	T	T	T
0.6096700000000000	0.4370100000000000	0.4721600000000002	T	T	T
0.2829000000000000	0.7778300000000000	0.6604500000000002	T	T	T
0.9074600000000002	0.8429700000000001	0.3071900000000001	T	T	T
0.5723300000000001	0.1778800000000000	0.4741400000000001	T	T	T
0.2328000000000000	0.5119500000000001	0.6546000000000001	T	T	T
0.5217300000000000	0.9989400000000002	0.3799400000000001	T	T	T
0.1909900000000000	0.3330300000000000	0.5477700000000001	T	T	T
0.8524700000000000	0.6674600000000001	0.7144900000000002	T	T	T
0.4100400000000000	0.8281300000000000	0.2780000000000001	T	T	T
0.0785800000000000	0.1594900000000000	0.4469500000000002	T	T	T
0.7435300000000000	0.4870200000000000	0.6185500000000003	T	T	T
0.3978900000000000	0.0618100000000000	0.3019100000000001	T	T	T
0.0615600000000000	0.4001900000000000	0.4725500000000001	T	T	T
0.7274500000000002	0.7332300000000000	0.6415600000000002	T	T	T
0.0083500000000000	0.7340200000000002	0.3705700000000001	T	T	T
0.6671800000000000	0.0580900000000000	0.5341500000000001	T	T	T
0.3333200000000001	0.4059700000000001	0.7059100000000001	T	T	T
0.7367600000000001	0.2351800000000000	0.3652900000000001	T	T	T
0.3974600000000000	0.5742699999999999	0.5346400000000001	T	T	T
0.0742100000000000	0.9126700000000001	0.7078200000000001	T	T	T
0.6723300000000001	0.6105200000000000	0.3019100000000001	T	T	T
0.3407200000000001	0.9455600000000001	0.4723600000000001	T	T	T
0.0120800000000000	0.2810600000000000	0.6399600000000001	T	T	T

0.7340800000000000	0.0000599999999999	0.3705700000000001	T	T	T
0.3985400000000000	0.3363200000000000	0.5382300000000001	T	T	T
0.0719300000000000	0.6740200000000001	0.7052200000000002	T	T	T
0.4919900000000000	0.7542300000000000	0.3944800000000001	T	T	T
0.1605800000000000	0.0864500000000000	0.5648200000000002	T	T	T
0.8217200000000000	0.4074800000000000	0.7298500000000002	T	T	T
0.5068300000000000	0.2716500000000000	0.3652900000000001	T	T	T
0.1748100000000000	0.6111600000000001	0.5358700000000001	T	T	T
0.8439500000000000	0.9359300000000002	0.7086100000000002	T	T	T
0.1654400000000000	0.0644900000000000	0.3071900000000001	T	T	T
0.8311700000000001	0.3984600000000000	0.4727000000000001	T	T	T
0.4985900000000000	0.7287100000000000	0.6596300000000003	T	T	T
0.9466000000000000	0.3360799999999999	0.3019100000000001	T	T	T
0.6082200000000000	0.6680200000000001	0.4706100000000002	T	T	T
0.2779700000000000	0.0004900000000000	0.6404200000000002	T	T	T
0.6627999999999999	0.8133500000000000	0.2925400000000001	T	T	T
0.3304000000000001	0.1443300000000000	0.4593100000000002	T	T	T
0.9941200000000001	0.4779300000000001	0.6288800000000002	T	T	T
0.2622300000000000	0.5164200000000000	0.3944800000000001	T	T	T
0.9297800000000001	0.8473900000000000	0.5651100000000002	T	T	T
0.5986500000000000	0.1887600000000000	0.7281700000000001	T	T	T
0.4772100000000000	0.4866800000000000	0.3799400000000001	T	T	T
0.1458600000000000	0.8181800000000001	0.5498400000000002	T	T	T
0.8243400000000000	0.1580500000000000	0.7217100000000002	T	T	T
0.4180900000000000	0.5983700000000000	0.2780000000000001	T	T	T
0.0847600000000000	0.9287500000000001	0.4475900000000000	T	T	T
0.7616700000000001	0.2505300000000000	0.6081700000000002	T	T	T
0.2743900000000000	0.2743300000000000	0.3705700000000001	T	T	T
0.9405000000000001	0.6083100000000001	0.5419000000000003	T	T	T
0.6084400000000000	0.9397100000000003	0.7030600000000002	T	T	T
0.1802800000000000	0.5903200000000000	0.2780000000000001	T	T	T
0.8488300000000000	0.9215700000000000	0.4468100000000000	T	T	T
0.5257300000000000	0.2740000000000000	0.6187300000000001	T	T	T
0.1505500000000000	0.3456100000000000	0.2925400000000001	T	T	T
0.8205600000000000	0.6775700000000000	0.4595900000000002	T	T	T
0.4903300000000000	0.0182600000000000	0.6273200000000002	T	T	T
0.1950600000000000	0.8578600000000001	0.2925400000000001	T	T	T
0.8653700000000001	0.1853700000000001	0.4573200000000001	T	T	T
0.5301000000000000	0.5232100000000000	0.6226500000000001	T	T	T
0.7732300000000001	0.5015800000000000	0.3652900000000001	T	T	T
0.4353900000000000	0.8308900000000001	0.5366400000000001	T	T	T
0.0995200000000000	0.1660900000000000	0.7078200000000002	T	T	T
0.5830700000000000	0.4437700000000000	0.7485900000000002	T	T	T
